# Methylglyoxal-Modified Human Serum Albumin Binds to Leukocyte Myeloperoxidase and Inhibits its Enzymatic Activity

**DOI:** 10.3390/antiox11112263

**Published:** 2022-11-16

**Authors:** Oleg M. Panasenko, Viktor A. Ivanov, Elena V. Mikhalchik, Irina V. Gorudko, Daria V. Grigorieva, Liliya Yu. Basyreva, Ekaterina V. Shmeleva, Sergey A. Gusev, Valeria A. Kostevich, Nikolay P. Gorbunov, Alexey V. Sokolov

**Affiliations:** 1 Department of Biophysics, Federal Research and Clinical Center of Physical-Chemical Medicine of Federal Medical Biological Agency, Moscow 119435, Russia; 2 Department of Medical Biophysics of the Institute for Translative Medicine, Pirogov Russian National Research Medical University, Moscow 117997, Russia; 3 Department of Biophysics, Belarusian State University, 220030 Minsk, Belarus; 4 Department of Molecular Genetics, Institute of Experimental Medicine, St. Petersburg 197376, Russia

**Keywords:** hyperglycemia, methylglyoxal, human serum albumin, myeloperoxidase, reactive oxygen species, reactive halogen species, neutrophil degranulation, NETosis

## Abstract

Hyperglycemia in diabetes mellitus induces modification of proteins by glucose and its derivative methylglyoxal (MG). Neutrophils perform their bactericidal activity mainly via reactive halogen (RHS) and oxygen (ROS) species generation catalyzed by myeloperoxidase (MPO) stored in neutrophil azurophilic granules (AGs) and membrane NADPH oxidase, respectively. Herein, we study the binding of human serum albumin (HSA) modified with MG (HSA-MG) to MPO and its effects on MPO activity and release by neutrophils. Peroxidase activity of MPO was registered by oxidation of 2,2′-azino-bis(3-ethylbenzothiazoline-6-sulfonic acid) diammonium salt, and chlorinating activity by decolorization of Celestine blue B dye. Binding of HSA-MG to MPO was studied by affinity chromatography, disc-electrophoresis, ligand Western blotting and enzyme-linked solid phase immunoassay using monoclonal antibodies (mAbs) to MPO. ROS and RHS generation were detected by lucigenin (Luc) and luminol (Lum) chemiluminescence (CL), respectively. Neutrophil degranulation was assessed by flow cytometry using fluorescent labeled antibodies to the marker proteins CD63 from AGs and CD11b from peroxidase-negative granules (PNGs). NETosis was assayed by quantifying DNA network-like structures (NET-like structures) in blood smears stained by Romanowsky. HSA-MG bound to MPO, giving a stable complex (K_d_ = 1.5 nM) and competing with mAbs, and non-competitively inhibited peroxidase and chlorinating MPO activity and induced degranulation of PNGs but not of AGs. HSA-MG enhanced Luc-CL per se or following PMA, unlike Lum-CL, and did not affect spontaneous or PMA-stimulated NETosis. Thus, HSA modified under hyperglycemia-like conditions stimulated NADPH oxidase of neutrophils but dampened their functions dependent on activity of MPO, with no effect on its release via degranulation or NETosis. This phenomenon could underlie the downregulation of bactericidal activity of MPO and neutrophils, and hence of innate immunity, giving rise to wound healing impairment and susceptibility to infection in patients with hyperglycemia.

## 1. Introduction

Methylglyoxal (MG) is a highly reactive α-ketoaldehyde generated in the human body as a by-product of a number of metabolic pathways and due to non-enzymatic glucose oxidation in hyperglycemia. MG is a potent protein glycating agent in numerous parallel and subsequent reactions collectively known as the Maillard reaction [1]. The key steps of the glycation process are (i) reaction of aldehydes with protein amino groups resulting in the formation of Schiff bases, (ii) transformation of the latter to more stable Amadori products and finally, (iii) formation of the advanced glycation end products (AGEs) [2]. Participation of MG in AGEs generation is accompanied by protein aggregation and formation of amyloid plaques, neurofibrillary clubs, cytoplasmic and intranuclear inclusions, which are features of pathologies such as diabetes mellitus, atherosclerosis, cataract, Alzheimer’s disease, etc. [2,3].

It is known that AGEs activate various cells via receptor-mediated mechanisms. Receptor for advanced glycation end products (RAGE) is the most studied receptor for AGEs on the cell surface, including neutrophils [4]. Receptor-mediated cellular activation proceeds via a number of signaling pathways [5]; in particular, the concentration of Ca^2+^ ions and actin polymerization are upregulated in neutrophil cytoplasm, NADPH oxidase is activated and N-formylmethionyl-leucyl-phenylalanine (fMLP)-induced generation of reactive oxygen species (ROS) is enhanced, as well as phagocytic activity. Paradoxically, an increase in the number of phagocytosed *Staphylococcus aureus* per neutrophil due to AGEs–receptor interactions was accompanied by an increase in bacterial viability in phagolysosomes [6].

Myeloperoxidase (MPO; EC 1.11.2.2) stored in neutrophil azurophilic granules (AGs) catalyzes the generation of reactive halogen species (RHS), which is a key factor of neutrophil antibacterial activity [7]. It was shown that exposure of neutrophils to AGEs stimulated MPO expression, depending on RAGE [8]. The paradoxical growth in phagocytic capacity and in MPO expression together with an increase in intercellular bacterial persistence in AGE-stimulated neutrophils made us hypothesize that AGEs could inhibit MPO enzymatic activity and hence the bactericidal function of neutrophils. Currently, there are no data on the effects of AGEs on MPO activity.

Our aim was to elucidate if the modification of human serum albumin (HSA) under hyperglycemia-like conditions affects enzymatic activity of MPO, its release from neutrophils by degranulation and NETosis, and the generation of ROS and RHS by neutrophils, which are key factors of neutrophil bactericidal activity.

For this purpose, HSA, the major protein of human blood plasma, was modified using one of the most reactive intermediates of glucose autoxidation, MG, modeling hyperglycemia-induced protein injury. The effects of MG-modified HSA (HSA-MG) on the MPO enzymatic activity and on the functional activity of neutrophils were studied.

## 2. Materials and Methods

### 2.1. Reagents Used

Human serum albumin (HSA), methylglyoxal (MG), D-glucose, Histopaque, sodium citrate, 3,3′,5,5′-tetramethylbenzidine (TMB), phorbol 12-myristate 13-acetate (PMA), 2,2′-azino-bis(3-ethylbenzothiazoline-6-sulfonic acid) diammonium salt (ABTS), celestine blue B (CB), 4-chloro-1-naphtol (4-CN), Coomassie G-250, *o*-dianisidine, hydrogen peroxide (H_2_O_2_), horseradish peroxidase (HRP), luminol (Lum), lucigenin (Luc), Triton X-100 and all salts and solvents for the preparation of solutions were purchased from Sigma-Aldrich (St. Louis, MO, USA). Acrylamide, methylene bis-acrylamide, tetramethylethylenediamine, ammonium persulphate, Tris and glycine were purchased from Panreac-AppliChem (Darmstad, Germany). Krebs-Ringer buffer solution was purchased from Merck (Kenilworth, NJ, USA). Anti-CD11b-PE, anti-CD63-APC, anti-CD45-FITC antibodies, non-fat dry milk (blotting grade blocker) and HRP-labeled anti-mouse IgG, nitrocellulose membrane were purchased from Bio-Rad (Hercules, CA, USA). Dextran T70 was purchased from Roth (Karlsruhe, Germany). May Grünwald’s Eosin–Methylene Blue solution and Romanowski Azur Eozin stain were purchased from ECOlab (Moscow, Russia).

### 2.2. Modification of HSA

HSA (10 mg/mL) was incubated with MG (100 mM) in 50 mM borate buffer, pH 8.6, over a period of 3 h to 7 days at 37 °C. Prior to incubation, the mixture of HSA with MG was passed through a 0.22 μm syringe filter (Corning, Kaiserslautern, Germany). HSA incubated without MG was used as a control. Then, the protein solution was washed thrice with 15-fold v/v Krebs-Ringer bicarbonate buffer using Amicon Ultra centrifugal filter (Merck, Kenilworth, NJ, USA) with 3 kDa cutoff, aliquoted and kept at −70 °C until use. Protein concentration was assessed by Lowry’s method. Modification degree was monitored by analysis of absorbance spectra (230–500 nm) on the spectrophotometer Cary 50 Bio (Varian, Mulgrave, Australia), by fluorescence emission spectra (λ_ex_ = 325 nm, λ_em_ = 350–500 nm) on the spectrofluorometer SOLAR CM 2203 (Solar, Minsk, Belarus) [9]. In all experiments, HSA-MG was prepared by 7-day-long modification, unless otherwise specified.

### 2.3. MPO Isolation

MPO was extracted from neutrophils of healthy volunteers by sonication on ice in 100 mM NaCl, 2 mM CaCl_2_, 10 mM Na-acetate buffer, pH 5.5, free of cationic detergents. Then, the affinity chromatography on heparin–Sepharose column, followed by hydrophobic chromatography on phenyl–agarose column and size-exclusion chromatography on Sephacryl S-200 HR column were performed, resulting in MPO preparation with *A*_430_/*A*_280_ (RZ) of 0.85, which corresponded to homogeneous enzymes. Hemi-MPO was obtained by treatment of purified dimeric MPO by 2-mercaptoethanol and iodoacetamide [10].

### 2.4. Anti-MPO Monoclonal Antibodies

Mice (BALB/c x DBA/2) F2 within 30 days were twice immunized subcutaneously into paw pads and withers with the MPO preparation obtained (50 μg per one animal); on day 12, the lymph nodes were extracted from mice, and the lymphocytes and myeloma were hybridized with SP2/0-AG14 cells according to the Köhler and Milstein technique [11]. The hybridomes were cultured in HAT-containing selective medium and after 2 weeks, the extracellularly produced antibodies were screened for their ability to bind to MPO immobilized in the solid phase, using ELISA with peroxidase-labeled anti-mouse IgG antibodies. Then, 100 µL of dimeric MPO (5 mg/L) in borate buffered saline (BBS: 150 mM NaCl, 10 mM sodium borate, pH 8.0) was dropped into the wells of standard polystyrene plate and incubated overnight at 4 °C, after which three washings were performed with BBS containing 0.05% Tween-20 (BBS-T, same washing performed before adding each reagent except termination of chromogenic reaction). Next, 200 µL of the blocking solution (3% milk in PBS, 0.05% Tween-20) was added into the wells. After 1 h incubation at 37 °C with continuous stirring at 300 rpm, the plate was washed with BBS-T, and 150 µL of the blocking solution and 50 µL of tested culture medium were added into each well. After 1 h incubation, 200 µL of the blocking solution containing HRP-labeled anti-mouse IgG (1:3000, Bio-Rad) was added into each well. Finally, 100 µL of a 0.5 mM TMB and 4 mM H_2_O_2_ mixture was added to each well. The reaction was terminated by adding 50 µL of 1 M H_2_SO_4_. The hybridization gave about 2000 clones, among which 20 clones producing anti-MPO monoclonal antibodies (mAbs) were selected. Later on, 6 clones were injected into the mouse peritoneal cavity to obtain a high yield of mAbs by the ascites method. Six mAbs were purified after salting out from ascites fluids with ammonium sulfate and subsequent chromatography on Protein A resin: 1#8, 2#7, 4#2, 4#4, 4#8, 4#10. Each mAb was conjugated with HRP using periodate oxidation of carbohydrate, followed by sodium borohydride reduction of Schiff base. According to sandwich ELISA of hemi-isoform of MPO with all possible combinations of solid-phase immobilized mAbs and HRP-labeled mAbs, all six mAbs interacted with different epitopes of MPO (see Table S1 in Supplementary Materials). Then, 100 µL of mAb (5 mg/L) in BBS was dropped into the wells of standard polystyrene plates and incubated overnight at 4 °C, followed by three washings with BBS-T. The same washing was performed before adding each reagent except for the termination of chromogenic reaction. Next, 100 µL of the blocking solution (3% milk in PBS, 0.05% Tween-20) was added into the wells. After 1 h incubation at 37 °C with continuous stirring at 300 rpm, the plate was washed with BBS-T, and 100 µL of the blocking solution containing 25, 50, 100 and 200 ng/mL of hemi-MPO was added into wells. After 1 h of incubation, 100 µL of the blocking solution containing 50 ng/mL of HRP-labeled mAb was added into each well. Finally, 100 µL of a 0.5 mM TMB and 4 mM H_2_O_2_ mixture was added to each well. The reaction was terminated by adding 50µL of 1 M H_2_SO_4_.

### 2.5. Neutrophil Isolation

Human neutrophils were isolated from 20 mL of blood of healthy donors stabilized with 109 mM sodium citrate solution (9:1 *v*/*v*). Blood was mixed with 6% dextran T70 in 155 mM NaCl at the 5:1 v/v ratio, and erythrocytes were allowed to sediment for 30–40 min in plastic tubes. The leukocyte-rich plasma (3–5 mL) was centrifuged for 7 min at 450 g and erythrocytes were removed by hypotonic lysis with 3 mL of cold 0.2% NaCl. Then, the cells were resuspended in 3 mL of 1.6% NaCl with 20 mg/mL glucose under gentle mixing. The cells were sedimented for 7 min at 450 g and resuspended in 6 mL of phosphate-buffered saline (PBS: 8.3 mM Na_2_HPO_4_; 1.2 mM KH_2_PO_4_; 123 mM NaCl; 2.7 mM KCl, pH 7.4), layered on top of 3 mL Histopaque 1.077 g/mL and subjected to centrifugation for 10 min at 450 g. Neutrophils were washed twice with PBS. All isolation procedures were performed at room temperature. The isolated neutrophils were suspended in PBS with 2 mg/mL glucose and kept at 4 °C for 3–4 h. The neutrophils comprised 97–98% in the resulted cell suspension, with viability of more than 96% according to trypan blue test.

For chemiluminescence (CL) assay, the neutrophils were isolated as follows: blood of healthy donors was collected into EDTA-vacutainers and then layered over the double gradient of Histopaque 1.077/1.119 g/mL. After centrifugation for 45 min, neutrophils were collected and washed with Krebs-Ringer solution.

Cell concentration was assayed by direct counting using a Goryaev chamber.

### 2.6. Affinity Chromatography of HSA-MG on MPO–Sepharose

MPO was immobilized from BrCN-activated Sepharose 6B (2.4 mg MPO per 1 mL of wet gel). After overnight incubation at 4 °C, residual active groups were blocked by 1 h incubation of resin with 49.4 mM Tris and 384 mM glycine, pH 8.3 [12]. MPO–Sepharose was packed into column (1.2 × 0.5 cm) and equilibrated by 150 mM NaCl, 10 mM Hepes-NaOH, pH 7.4 (HBS). Then, 1 mL of 10 μM HSA or HSA-MG was loaded on column, followed by elution with 6 mL of equilibration buffer and 6 mL of 1 M NaCl, 10 mM Hepes-NaOH, pH 7.4, and the collection of 1 mL fractions for further analysis was performed. Collected fractions were analyzed by protein content, *A*_280_ and MPO-binding capacity (ELISA and ligand Western blotting assay).

### 2.7. Detection of MPO and HSA-MG Interaction Using Disc-Electrophoresis without Detergent and Ligand Western Blotting Assay

Briefly, 5% polyacrylamide gel with 125 mM Tris–HCl, pH 6.8, was used as a stacking one, and 7.5% polyacrylamide gel with 375 mM Tris–HCl, pH 8.8, was used as a resolving one. To the mixture of buffer solution and acrylamide (acrylamide/methylene bis-acrylamide = 30%/0.8%), tetramethylethylenediamine and ammonium persulphate were added, each up to 0.1%. Then, 4.94 mM Tris with 38.4 mM glycine, pH 8.3, was used as an electrode buffer. MPO, HSA and HSA-MG samples (5–15 µg of protein) in various combinations as well as fractions of HSA/HSA-MG obtained from MPO–Sepharose were mixed in the ratio 4:1 (*v*/*v*) with 62.5 mM Tris–HCl buffer, pH 6.8, containing 0.001% bromophenol blue and 50% glycerol, and loaded into the wells of the stacking gel. Electrophoresis was carried out for 2.5–3 h at a potential gradient of 15–20 V/cm (2–5 mA/cm of gel) in the vertical electrophoresis cell. Bromophenol blue dye served as a marker of run end. Gels were stained to detect MPO activity (with chromogenic mixture of 0.2 mM *o*-dianisidine and 0.1 mM H_2_O_2_ in 0.1 M sodium acetate buffer solution, pH 5.5, giving orange-brown bands, with subsequent washing with distilled H_2_O) or to detect protein (after fixation in 25% ethanol for 10 min, a mixture of 0.5% Coomassie G-250 in 25% ethanol and 25% H_3_PO_4_ was applied for 10 min with subsequent washing with 25% ethanol). The resulting gels were scanned. For ligand Western blotting assay, proteins separated in gel were transferred to nitrocellulose membrane by semi-dry apparatus, followed by 15 min blocking in 3% milk in PBS, containing 0.05% Tween 20. In the next step, the membrane was incubated for 1 h with 1 μg/mL MPO in blocking solution, and after washing thrice for 5 min with PBS with 0.05% Tween 20, the membrane was incubated for 1 h with HRP-labeled anti-MPO mAb 1#8 (300 ng/mL) in blocking solution. After washing thrice for 5 min with PBS with 0.05% Tween 20, the membrane was stained with 4-CN and H_2_O_2_ and washed with water, and scanned after purple color development.

### 2.8. Detection of MPO and HSA-MG Interaction by ELISA

Briefly, 100 µL per well of HSA-MG at 2 µg/mL in borate buffer solution BBS was loaded into a 96-well plate and incubated in a thermo-shaker for 2 h at 37 °C, 350 rpm. Then, the wells were washed with BBS-T, and 100 µL of 3% skim milk in BBS-T containing MPO was added (0–500 ng/mL). After 1 h incubation in a thermo-shaker (at 37 °C and 350 rpm), the plate was washed with BBS-T, and 100 µL of 3% skim milk in BBS-T containing 200 ng/mL HRP-labeled anti-MPO mAbs was added: HRP-1#8; HRP-2#7, HRP-4#2, HRP-4#4, HRP-4#8, HRP-4#10. After 1 h incubation in a thermo-shaker (at 37 °C, 350 rpm), the plate was washed with BBS-T, and 100 µL of a 0.5 mM TMB and 4 mM H_2_O_2_ mixture was added to each well. The reaction was stopped by adding 50 µL of 1 M H_2_SO_4_.

### 2.9. Evaluation of Dissociation Constant (K_d_) Value of MPO-HSA-MG Complex and MPO-Binding Capacity Assay

Briefly, 100 µL per well of HSA-MG or HSA at 2 µg/mL, or anti-MPO mAbs 2#7 at 5 µg/mL in BBS, was loaded into a 96-well plate and incubated in a thermo-shaker for 3 h at 37 °C, 350 rpm. Then, the wells were washed with BBS-T, and 100 µL of 3% skim milk in BBS-T containing MPO was added (0.1–800 ng/mL to HSA-MG-wells, and 0.782–800 ng/mL MPO to the wells containing 2#7 mAbs). After 1 h incubation in a thermo-shaker (at 37 °C and 35 rpm), the plate was washed with BBS-T, and 100 µL of a 0.5 mM TMB and 4 mM H_2_O_2_ mixture was added to each well. The reaction was stopped by adding 50 µL of 1 M H_2_SO_4_. Absorbance at 450 nm was read and plotted versus MPO concentration in the absence and presence of 2#7 mAbs. The concentration of MPO bound to HSA-MG, the concentration of free MPO (as a difference between MPO added and bound), as well as the ratio of bound MPO and free MPO (B/F) were calculated. The results are presented using Scatchard coordinates. The linear regression equation was computed as [B]/[F] = 1/K_d_ − [B]/K_d_, and K_d_ was calculated corresponding to the slope of the line. Minor modification of protocol was performed for testing the MPO-binding capacity of HSA-MG or HSA fraction obtained from affinity chromatography on MPO–Sepharose. Then, 500 ng/mL of the tested protein was immobilized and 2000 ng/mL MPO samples were added in the next step for testing the binding of MPO. Specific MPO-binding capacity of the tested protein (ng/μg) was calculated to level of protein immobilized in wells.

### 2.10. MPO Enzymatic Activity

Peroxidase activity of MPO in the presence of HSA or HSA-MG was assayed using the chromogenic substrate ABTS. Oxidized by Compounds I and II of MPO, it is converted to a stable radical, green in color. The rate of oxidation can be easily assessed by *A*_414_ and ε_414_ = 36,000 M^−1^cm^−1^. The reaction was started with the addition of H_2_O_2_ and the initial rate was registered as Δ*A*_414_/min. Each point was evaluated in triplicate, and the rate was expressed in nM ABTS per s using ε_414_ and normalized by enzyme concentration (nM). In order to evaluate K_M_ for H_2_O_2_, its concentration was varied from 2 μM to 50 µM in the mixture containing 5 nM MPO (with or without 500 nM HSA or HSA-MG) and 1 mM ABTS in 50 mM Na-acetate buffer, pH 5.5. In order to evaluate K_M_ for ABTS, its concentration was varied from 0.4 mM to 2 mM in the mixture containing 5 nM MPO (with or without 500 nM HSA or HSA-MG) and 50 µM H_2_O_2_ in 50 mM Na-acetate buffer, pH 5.5. For the samples containing 5 nM MPO, 5 nM MPO and 500 nM HSA, and 5 nM MPO and 500 nM HSA-MG, the Hanes–Woolf plots were made as [H_2_O_2_]/V = f([H_2_O_2_]) and [ABTS]/V = f([ABTS]). K_M_ was equal in magnitude to the intercept made on an abscissa axis by the approximating straight line. V_max_ was calculated as a reciprocal value of the straight-line slope. In the control experiment, the rate of oxidation of 1 mM ABTS in the presence of 2 mM H_2_O_2_ by 5 nM HRP (with or without 500 nM HSA HSA-MG) in 50 mM Na-acetate buffer, pH 5.5, was tested.

Chlorinating activity of MPO in the presence of HSA or HSA-MG was assessed by decolorization of CB caused by its reaction with HOCl and taurine chloramine generated by MPO at the rate, which can be expressed as a decrease in CB optical absorbance. The reaction was initiated by adding H_2_O_2_, and the initial decolorization rate was registered as -Δ*A*_650_/min. At each point, the measurements were carried out in triplicate. The average rate values were converted to nM of HOCl per s using ε_650_ = 15,200 M^−1^cm^−1^ for CB [13] and normalized by enzyme concentration (10 nM). In order to determine K_M_ for H_2_O_2_, its concentration was varied from 5 µM to 80 µM in the mixture containing 10 nM MPO (with or without 500 nM HSA or HSA-MG), 150 mM NaCl, 200 µM CB, 2 mM taurine, 10 µM KI in 20 mM Na-phosphate buffer, pH 5.8. In order to determine K_M_ for NaCl, its concentration was varied from 25 mM to 150 mM in the mixture containing 10 nM MPO (with or without 500 nM HSA or HSA-MG), 50 µM H_2_O_2_, 200 µM CB, 2 mM taurine, 10 µM KI in 20 mM Na–phosphate buffer, pH 5.8. For the samples containing 10 nM MPO, 10 nM MPO and 500 nM HSA, and 10 nM MPO and 500 nM HSA-MG, the Hanes–Woolf plots were made as [H_2_O_2_]/V = f([H_2_O_2_]) and [NaCl]/V = f([NaCl]). K_M_ was equal in magnitude to the intercept made on an abscissa axis by the approximating straight line. V_max_ was calculated as a reciprocal value of the straight-line slope. In the control experiment, the oxidation of 200 μM CB by 25 μM HOCl (with or without 500 nM HSA or HSA-MG) in 150 mM NaCl, 2 mM taurine, 10 µM KI in 20 mM Na-phosphate buffer, pH 5.8, was tested.

The inhibition constant (K_i_) for noncompetitive inhibition was calculated as follows: K_i_ = [I]/((V_max_ − V_max_’)/V_max_’), where [I] is the concentration of inhibitor, V_max_ is the maximal rate without inhibitor and V_max_’ is the maximal rate in the presence of inhibitor.

### 2.11. Neutrophil Chemiluminescence

Lucigenin- or luminol-dependent chemiluminescence (Luc-CL or Lum-CL) of isolated neutrophils was measured with the luminometer Lum1200 (DiSoft, Moscow, Russia). Neutrophils (0.4 × 10^6^ cells/mL) were added into Krebs-Ringer solution (pH 7.4) with 0.1 mM Luc or 0.2 mM Lum and spontaneous CL was registered for some time; then, HSA or HSA-MG was added up to 1 mg/mL and the CL kinetics were registered until maximum values were reached. The CL amplitude (V) was calculated as the difference between maximum and spontaneous values. Then, the cells were stimulated with PMA (0.16 µM) and the CL kinetics were registered until maximum values were reached.

### 2.12. Assays for Degranulation of Human Neutrophils

To prepare samples for flow cytometry, the neutrophils (1 × 10^6^ cells/mL in PBS supplemented with 1 mM CaCl_2_ and 0.5 mM MgCl_2_) were exposed to HSA or HSA-MG in various concentrations for 10 min at 37 °C. Then, neutrophils were treated with anti-CD11b-PE and anti-CD63-APC (1% *v*/*v*, incubation for 5 min at room temperature). The resulting cell suspension was analyzed using a CytoFLEX Flow Cytometer (Beckman Coulter, Brea, CA, USA) to quantify CD11b- or CD63-positive cells in a selected neutrophil population based on forward and side scatter characteristics and anti-CD45-FITC fluorescence intensity. The experimental results were processed using CytExpert 2.4 software. At least 20,000 neutrophils were analyzed in each sample.

Degranulation of AGs was also assayed by MPO secretion. Neutrophils (2.3 × 10^6^ cells/mL) were incubated with 1 mg/mL HSA or HSA-MG in bicarbonate Krebs-Ringer solution with CaCl_2_, pH 7.4, supplied with 2% autologous blood plasma, for 30 min at 37 °C. Then, 150 mM NaCl was added to the cells as a negative control, and 1 mg/mL zymosan as a positive control. The cells were sedimented by centrifugation at 400× *g* for 10 min, and cell-free supernatant fluid was aspirated—sample “1”. The cells were washed with 2 mL of 150 mM NaCl and lysed with 0.25 mL of 1% Triton X-100. The cell lysates were resedimented for 10 min at 900× *g*, and supernatant fluid was aspirated—sample “2”. Then, peroxidase MPO activity was measured in the supernatant fluids sample “1” (extracellular content) and sample “2” (intracellular content) with *o*-dianisidine [14]. In preliminary experiments, the concentration of MPO was assayed using ELISA kit (Cloud-Clone Corp., Wuhan, China)—see Figure S1 in Supplementary Materials.

### 2.13. Spontaneous and PMA-Stimulated NETosis

EDTA-stabilized capillary blood of four healthy volunteers was obtained via a finger prick. In order to evaluate spontaneous NETosis, the blood aliquots of 20 µL were placed into 1.5 mL Eppendorf tubes and mixed with 0.5 mg/mL or 1 mg/mL of HSA or HSA-MG modified for a period of 48 h, and incubated for 3 h at 37 °C. When PMA-stimulated NETosis was assayed, after the first 1 h, PMA was added up to 100 nM to a number of samples, and the incubation continued for another 2 h at gentle tuning up and down with a Multi Bio RS-24 rotator (Biosan, Riga, Latvia). Samples without HSA or HSA-MG served as a negative control. Counting of neutrophil extracellular traps (NET-like structures) was performed using standardized blood smears stained by the Romanowsky method. Three to five smears were prepared from each blood sample. The smears were examined using a Motic B3 microscope (Motic Asia, Hong Kong). NET-like structures per 300–500 leukocytes were counted in the middle third of the smear, and the NET-to-leukocyte percentage ratio was calculated [15]. DNA in NET-like structures was visualized with Hoechst 33,342 staining (see Figure S2 in Supplementary Materials) using the Nikon Eclipse Ni-E fluorescent microscope (Nikon, Tokyo, Japan). The leukocyte count was evaluated in a Goryaev chamber.

### 2.14. Statistical Analysis

All data are expressed as the mean ± SEM. Comparisons between two groups were made by Student’s *t*-test (2-tailed) and one-way ANOVA with Tukey’s post hoc analysis for between-group analysis involving more than two groups. A difference between the groups of *p* < 0.05 was considered significant.

## 3. Results

### 3.1. Characteristics of HSA-MG

**Figure 1:**
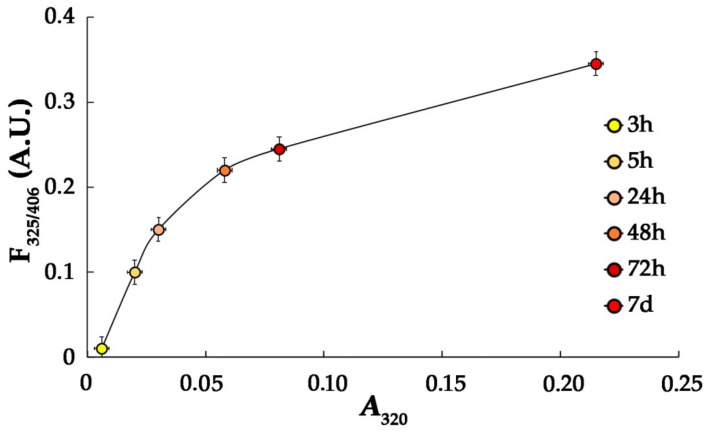
Plot of fluorescence (λ_ex_/λ_em_ = 325/406 nm) versus optical absorbance at 320 nm for HSA-MG at various durations of modification (indicated in the figure). For standard HSA solution A_320_ = 0.003; F_325_/F_406_ = 0. All measurements were performed in triplicate (*n* = 3).

The growth of optical absorbance of protein solution at 280 nm and 320 nm, an increase in fluorescence intensity at 406 nm (λ_ex_ = 325 nm) and partial HSA polymerization according to the data of gel-filtration were detected in the time-course of HSA modification with MG, being consistent with data of other researchers [9]. As is shown in Figure 1, optical absorbance of solution at 320 nm correlated with fluorescence (λ_ex_ = 325 nm, λ_em_ = 406 nm). Optical absorbance at 320 nm and fluorescence at λ_em_/λ_ex_ = 325/406 nm are attributed to AGEs; these results confirm their formation during incubation of HSA with MG [9].

### 3.2. HSA-MG Binding with MPO

**Figure 2:**
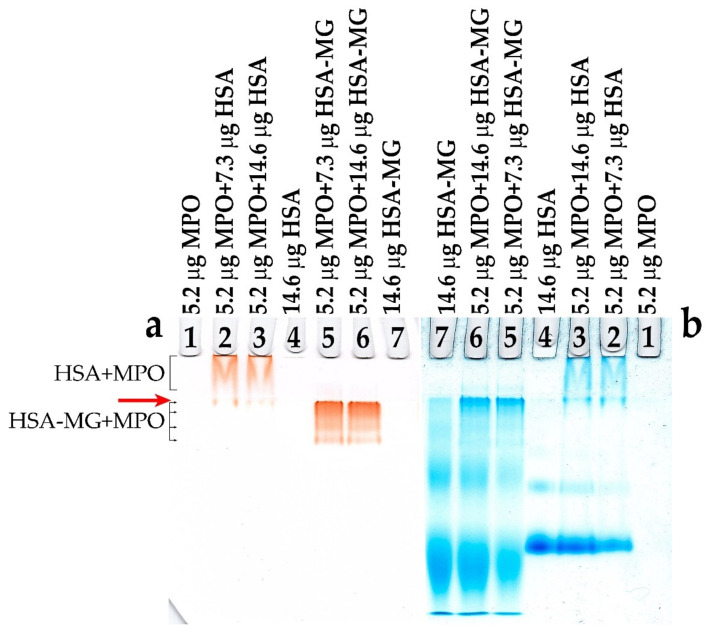
Disc-electrophoresis in polyacrylamide gel without detergents of samples containing MPO, HSA or HSA-MG (1:100, mol/mol). (**a**) Left gel stained by peroxidase activity (0.2 mM *o*-dianisidine and 100 μM H_2_O_2_, 100 mM Na-acetate buffer, pH 5.5). (**b**) Right gel stained by protein-specific dye—Coomassie G-250. Red arrow marked joining between stacking and resolving gels, positions of HSA + MPO and HSA-MG+MPO bands marked by black brackets. 1—MPO (36 pmol—5.2 µg), 2—MPO (36 pmol—5.2 µg) + HSA (110 pmol—7.3 µg), 3—MPO (36 pmol—5.2 µg) + HSA (220 pmol—14.6 µg), 4—HSA (220 pmol—14.6 µg), 5—MPO (36 pmol—5.2 µg) + HSA-MG (110 pmol—7.3 µg), 6—MPO (36 pmol—5.2 µg) + HSA-MG (220 pmol—14.6 µg), 7—HSA-MG (220 pmol—14.6 µg).

Binding of HSA-MG with MPO was assayed by changes in the electrophoretic mobility of the proteins demonstrated by disc-electrophoresis in polyacrylamide gel without detergents. Figure 2 shows the electropherogram after disc-electrophoresis of the samples, containing MPO, HSA and HSA-MG, with gel staining for MPO activity (Figure 2a) and total protein (Figure 2b).

Being a protein with high pI~9–10, MPO does not migrate through the gel because of MPO’s positive charge, even at pH 8.3 of the electrode buffer solution, and hence, no protein and peroxidase activity were detected in lane 1 (Figure 2a,b).

When HSA was present in the mixture in excess, the upper part of the gel (Figure 2a, lanes 2 and 3) showed a V-shaped band of staining peroxidase activity, which witnessed weak protein–protein interaction. It is noteworthy that at the end of electrophoresis, substantially all HSA migrated to the lower part of the resolving gel, while MPO remained entirely in the stacking gel and did not overcome its board. Modification of HSA with MG resulted in a notable change in its charge, enhanced protein dimerization and aggregation detected as a shift in the major band to anode, an increase in the intensity of staining of the HSA dimer band and the appearance of staining on the boundary between the stacking and resolving gels (Figure 2b, lane 7). When HSA-MG was added to MPO, a new pattern formed consisting of at least three bands below the board between the stacking and resolving gel (marked by red arrow). Such patterns argue for the generation of multicomponent complexes including polymers of HSA-MG and dimeric MPO (Figure 2b, lanes 5 and 6).

**Figure 3:**
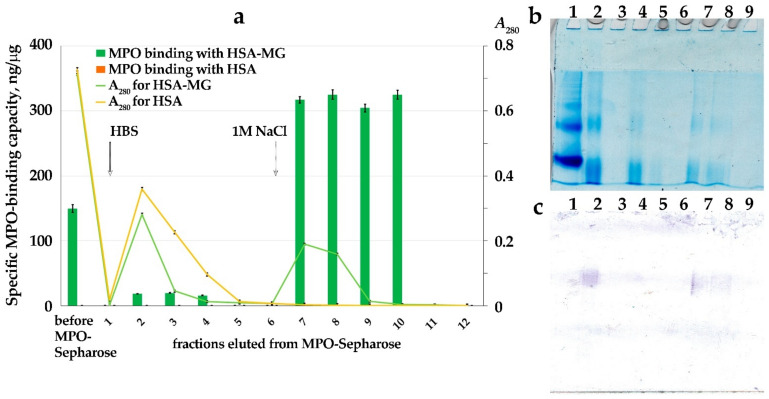
Analysis of fractions obtained by affinity chromatography of 1 mL 10 μM HSA or HSA-MG on MPO–Sepharose. (**a**) Profile of elution (*A*_280_) and specific MPO-binding capacity of protein in fractions. Start of elution by HBS (fractions 1–6) and by 1 M NaCl (fractions 7–12) marked by arrows (*n* = 3). (**b**) Resolution of proteins by disc-electrophoresis in polyacrylamide gel without detergents of 20 μL samples of loaded protein HSA (1), HSA-MG (2), fractions 1–4 (3–6) and 7–9 (7–9) of HSA-MG. Stained by protein-specific dye—Coomassie G-250. (**c**) Ligand Western blotting assay of same gel (as in (**b**)) after incubation with 1 μg/mL MPO, 300 ng/mL HRP-1#8 (anti-MPO), followed by 4-CN with H_2_O_2_ staining.

The pattern of HSA-MG interacting with MPO was studied using affinity chromatography of both modified and control proteins (Figure 3). Following application of 1 mL of 10 µM HSA or HSA-MG on the column with 1 mL of MPO–Sepharose, six 1 mL fractions eluted with HBS and another six 1 mL fractions eluted with 1 M NaCl were collected. Measurement of specific MPO-binding capacity and analysis of MPO binding using ligand Western blotting have shown that about half of the HSA-MG applied on the column remained bound to sorbent and could be fully eluted with 1 M NaCl (Figure 3a).

It is to be noted that specific MPO-binding capacity was not detected in fractions eluted with HBS, while it increased twice in fractions eluted with 1 M NaCl. In control experiments with HSA, no binding of the protein to sorbent was detected, and it also did not bind to MPO according to ELISA and ligand Western blotting data.

Ligand Western blotting (Figure 3c) has shown that it was mainly the dimeric form of HSA-MG that migrated in the middle of the resolving gel to bind with MPO. There was no significant binding of MPO with the zone corresponding to monomeric HSA and HSA-MG.

**Figure 4:**
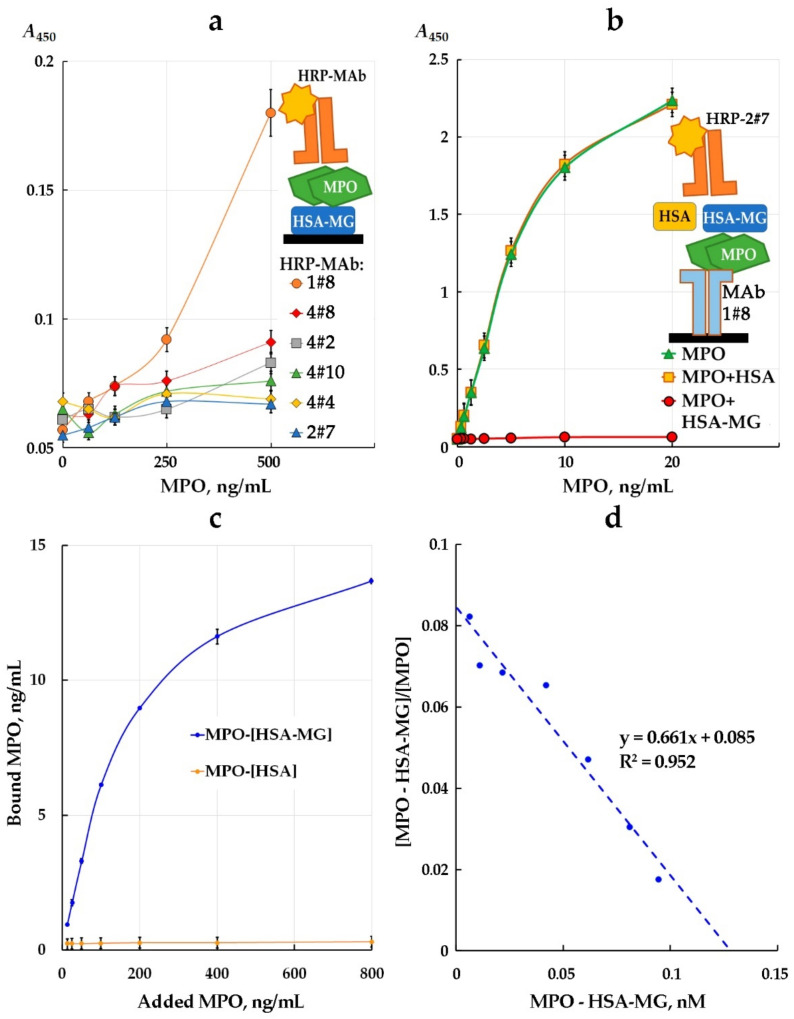
Illustration of competition between HSA-MG and mAbs against MPO for binding MPO in ELISA and determination of K_d_ of complex MPO with HSA-MG. (**a**) Competition in case of HSA-MG immobilization on solid phase, detection of MPO by HRP-labeled mAbs with TMB-H_2_O_2_-staining (*A*_450_). (**b**) Competition in case of adding HSA-MG (or HSA—negative control) to MPO samples in “sandwich” ELISA: mAbs (1#8) immobilization on solid phase, detection of MPO by HRP-labeled mAbs (2#7) with TMB-H_2_O_2_-staining (*A*_450_). (**c**) Binding of MPO with HSA-MG or HSA in ELISA assay. (**d**) Scatchard plot illustrating dependence of [MPO-HSA-MG]/[MPO]: ratio of MPO in complex with HSA-MG to free MPO from concentration of complex MPO-HSA-MG. All measurements were performed in triplicate (*n* = 3).

The assay of interaction between MPO and HSA-MG by ELISA included sorption of HSA-MG onto the surface of polystyrene plates followed by blocking of non-specific binding, then the addition of various concentrations of MPO followed by HRP-labeled anti-MPO mAbs. Among six tested mAbs, only 1#8-HRP could reach an increase in the signal of peroxidase label depending on the concentration of MPO added (Figure 4a).

Moreover, it was impossible to detect MPO in the mixture with HSA-MG using sandwich-type ELISA, when one of the mAbs was immobilized onto the solid surface and another mAb labeled with HRP was used to detect MPO bound to the first one.

In order to evaluate the constant of dissociation K_d_, HSA-MG was adsorbed onto the solid phase and adsorbed mAbs 2#7 was used as a control. Various concentrations of MPO were then added, and after washing of the plate, the amount of HSA-MG-bound MPO was measured using mAbs 1#8-HRP (Figure 4c). The same experiment with control HSA did not show any binding of MPO with HSA in such conditions. From the difference between added and bound MPO, the free MPO concentration was calculated, and the results are presented using Scatchard coordinates (Figure 4d). According to the slope of the line, K_d_ was equal to 1.5 nM. Such a low value of K_d_ is characteristic of a highly affine interaction, taking into account non-equilibrium conditions after plate washing and at the stage of binding of mAbs 1#8-HRP to detect MPO. Thus, the interaction of MPO with HSA-MG showed affinity comparable with antigen-antibody affinity detected by ELISA.

### 3.3. Effects of HSA-MG on Enzymatic Activity of MPO

**Figure 5:**
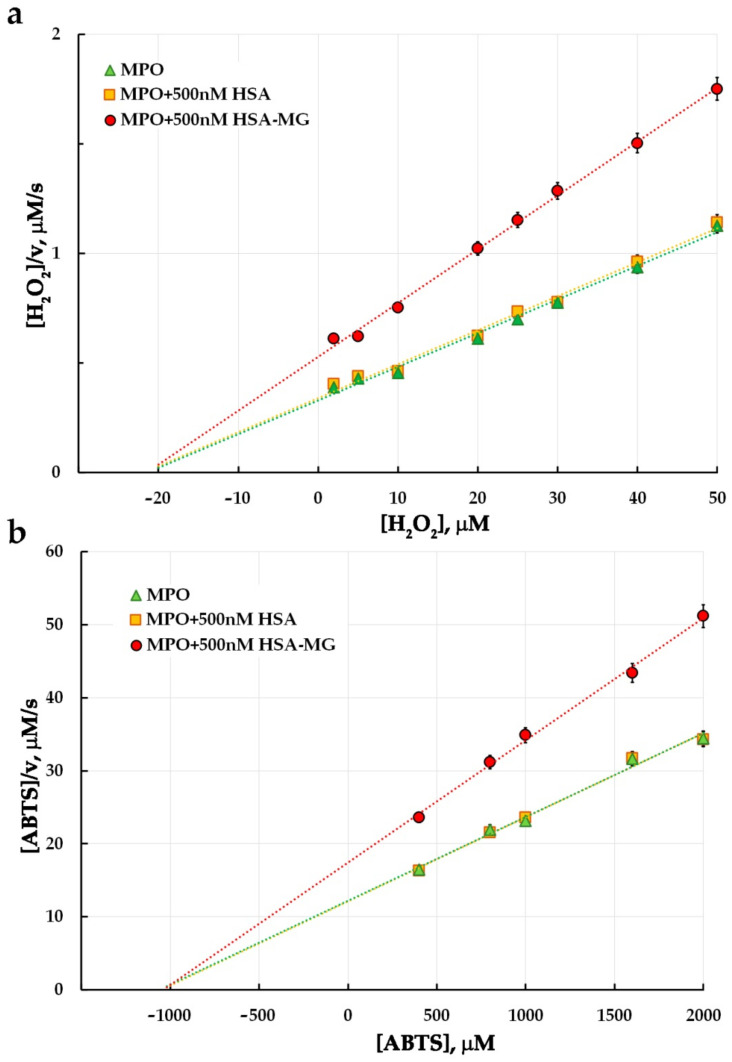
Hanes–Woolf plots illustrating the effect of HSA-MG on kinetics of MPO-catalyzed ABTS oxidation (*n* = 3). (**a**) The mixture contained 1 mM ABTS, 5 nM MPO, 2–50 µM H_2_O_2_ in 50 mM sodium acetate buffer, pH 5.5; HSA or HSA-MG were added to achieve concentration of 0.5 µM. (**b**) The mixture contained 50 µM H_2_O_2_, 5 nM MPO, 0.4–2.0 mM ABTS in 50 mM sodium acetate buffer, pH 5.5; HSA or HSA-MG were added to achieve concentration of 0.5 µM.

**Figure 6:**
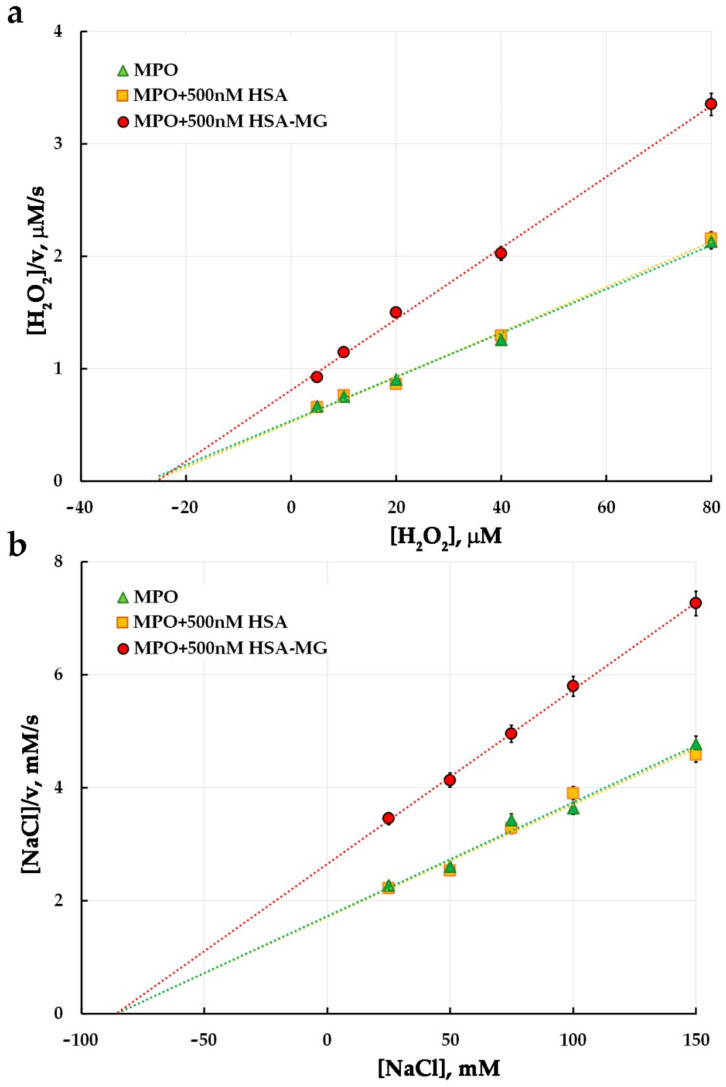
Hanes–Woolf plots illustrating the effect of HSA-MG on kinetics of MPO-catalyzed chloride oxidation (*n* = 3). (**a**) The mixture contained 150 mM NaCl, 10 nM MPO, 200 µM CB, 2 mM Tau, 10 µM KI, 5–80 µM H_2_O_2_ in 20 mM sodium–phosphate buffer, pH 5.8; HSA or HSA-MG were added to achieve concentration of 0.5 µM. (**b**) The mixture contained 50 µM H_2_O_2_, 10 nM MPO, 200 µM CB, 2 mM Tau, 10 µM KI, 25–150 mM NaCl in 20 mM sodium–phosphate buffer, pH 5.8; HSA or HSA-MG were added to achieve concentration of 0.5 µM.

The results of the evaluation of the HSA-MG effects on MPO peroxidase and chlorinating activity are represented in Hanes–Woolf coordinates (Figure 5; Figure 6, respectively). In the control experiment, the rate of oxidation of 1 mM ABTS in the presence of 2 mM H2O2 by 5 nM HRP as well as oxidation of 200 mM CB by 25 mM HOCl, with or without 500 nM HSA or HSA-MG did not change (see Table S2 in Supplementary Materials).

It is apparent that non-modified HSA does not affect ABTS or CB oxidation by HOCl and hence does not influence peroxidase or chlorinating activity of MPO. Unlike HSA, HSA-MG significantly inhibited both peroxidase and chlorinating enzyme activity. The values of K_M_ and V_max_, found as the line slope in Hanes–Woolf plots, and K_i_ values are represented in Table 1.

From the effects of HSA-MG on K_M_ and V_max_ of oxidation of substrates for peroxidase and chlorinating activity of MPO, one can see that neither for ABTS nor for H_2_O_2_ or NaCl were the values of K_M_ influenced. At the same time, HSA-MG significantly decreased the V_max_ value for all these substrates, suggesting a non-competitive mechanism of MPO inhibition. K_i_ values ranged from 0.80 µM to 1.08 µM, while K_i_ per se could be considered as a dissociation constant of an enzyme inhibitor complex, MPO-HSA-MG.

It is noteworthy that not all of the MG-modified HSA molecules (visualized as various patterns of HSA-MG in Figure 2; Figure 3) cause changes influencing the functionality of the enzyme’s active site. That is why the calculated K_i_ value appeared much greater than the K_d_ value. The found K_i_ value is indicative of more affinity to MPO of HSA-MG compared to non-modified HSA, for which the K_d_ of its complex with MPO was evaluated earlier as 20 ± 1.5 µM [16].

### 3.4. Effects of HSA-MG on Activation of Neutrophils

**Figure 7:**
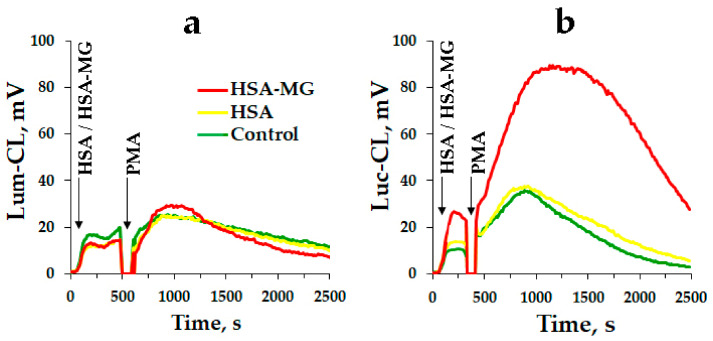
Typical time-courses of Lum-CL (**a**) and Luc-CL (**b**) response of neutrophils to non-modified HSA or HSA-MG with subsequent addition of PMA. Aliquots of 150 mM NaCl were added instead of protein solutions to the control samples. Arrows indicate moments of HSA/HSA-MG and PMA addition. The measurements were carried out at 37 °C in bicarbonate Krebs-Ringer buffer solution (pH 7.4) supplied with 1.3 mM CaCl_2_, 0.2 mM Lum or 0.1 mM Luc. Each probe contained 0.4 × 10^6^ cells/mL; 0.25 mg/mL HSA or HSA-MG; 0.16 µM PMA.

Neutrophil activation was registered by the method of Lum-CL or Luc-CL. Figure 7a,b represents the typical time-course of the neutrophil CL response to HSA or HSA-MG followed by PMA addition. Obviously, the time-course of Lum-CL was not influenced by HSA or HSA-MG compared to the control probes, neither before nor after neutrophil stimulation with PMA (Figure 7a). At the same time, in the presence of HSA-MG, Luc-CL grew about twofold in intensity compared to the control probe or HSA, and PMA-stimulated Luc-CL also significantly increased (Figure 7b).

### 3.5. Effects of HSA-MG on Degranulation of Neutrophils

**Figure 8:**
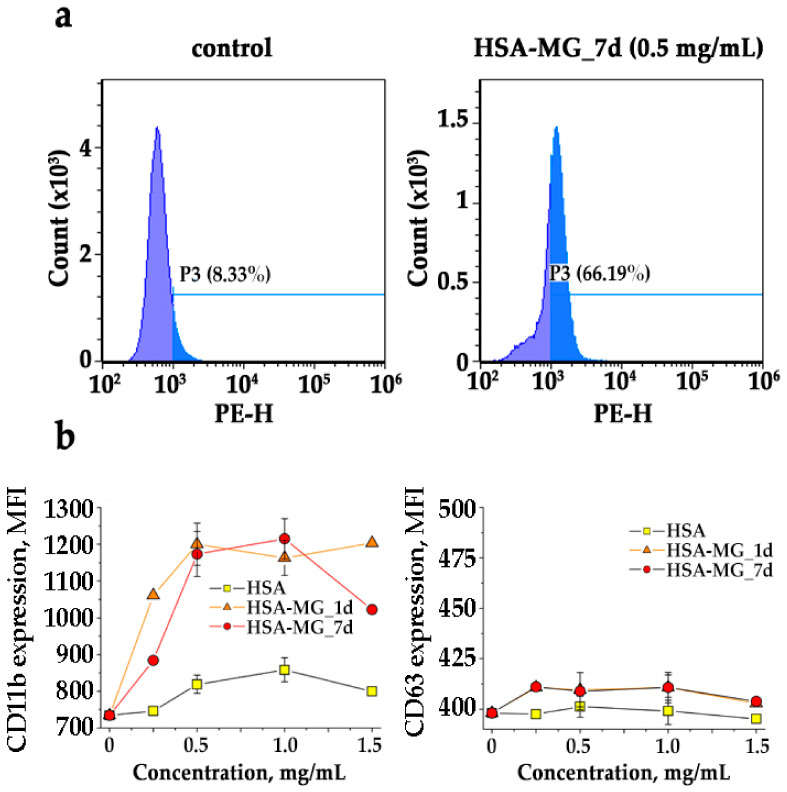
The effect of HSA-MG on neutrophil degranulation. (**a**) Histogram of fluorescence intensity of CD11b-PE bound to neutrophils in the absence (control) and presence of HSA-MG (0.5 mg/mL) (for PE fluorescence excitation—488 nm, emission—585 ± 42 nm). Data are shown for one of three independent experiments. (**b**) Expression of CD11b (marker of PNGs, left) and CD63 (marker of AGs, right) on plasma membrane of neutrophils incubated with native HSA, HSA-MG_1d (HSA was incubated with MG ap to 24 h) and HSA-MG_7d (HSA was incubated with MG ap to 7 days) in different concentrations. For APC fluorescence, excitation was 638 nm, emission was 660 ± 10 nm. Experiments were carried out in Ca^2+^-containing PBS, and at least 20,000 neutrophils were analyzed in each sample. Results are expressed in mean fluorescence intensity (MFI) ± SEM of at least three independent experiments.

Neutrophil degranulation was analyzed by flow cytometry. Exposure of CD63 (a marker of AGs and CD11b (a marker of peroxidase-negative specific/gelatinase granules (PNGs)) was detected using the fluorescent labeled antibodies (Figure 8a,b). It has been found that neutrophils preincubated with HSA-MG without any fluorescent label showed no fluorescence in channels for the APC and PE registration. After incubation of neutrophils with HSA-MG (with a duration of HSA modification of 24 h and 7 d), the dose-dependent increase in CD11b expression on the cell surface was registered (Figure 8b), indicating exocytosis of the contents of PNGs. The maximal effect was reached at 0.5 mg/mL HSA-MG.

**Figure 9:**
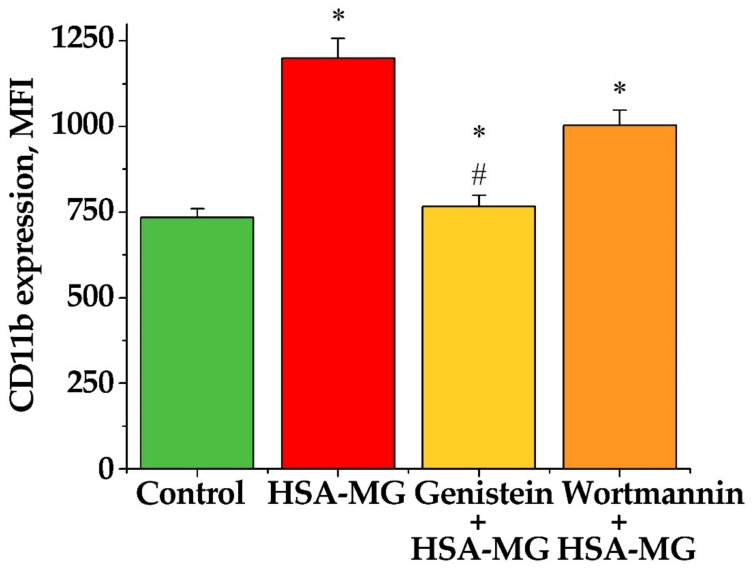
Effect of genistein (100 μM) and wortmannin (100 nM) on expression of CD11b on neutrophil surface in response to HSA-MG (0.5 mg/mL). Results are expressed in mean fluorescence intensity (MFI) ± SEM of at least three independent experiments. * *p* < 0.05 compared to control, # *p* < 0.05 compared to HSA-MG effect.

Neutrophil preincubation with 100 µM genistein partially (by ~70%) canceled the HSA-MG-stimulated CD11b exposure (Figure 9). A specific inhibitor of phosphoinositide-3-kinases, wortmannin (100 nM), inhibited HSA-MG-induced degranulation of neutrophils only by ~20% (Figure 9). These results are in agreement with findings that genistein, a broad specificity tyrosine kinase inhibitor, blocked the exocytosis of primary and secondary granules [17] and wortmannin inhibits the exocytosis of secondary granule contents of neutrophils [18].

**Figure 10:**
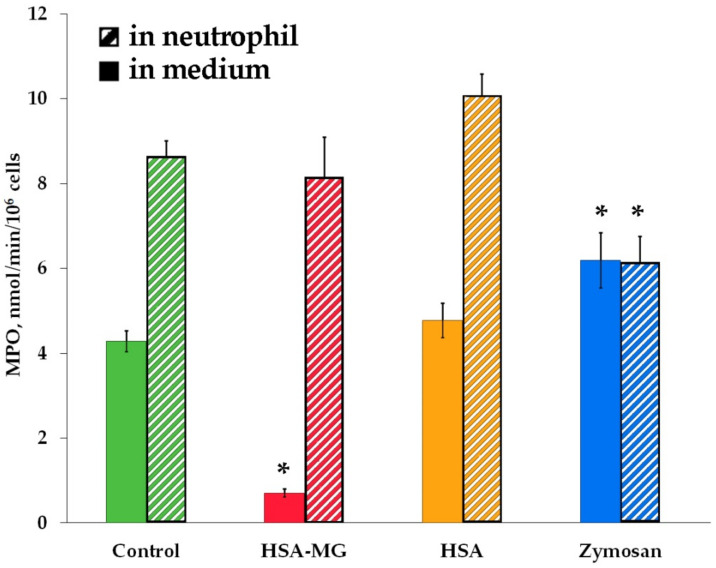
Level of MPO in neutrophils and in extracellular medium after cell incubation with HSA or HSA-MG compared with zymosan. Cells were incubated in bicarbonate Krebs-Ringer buffer solution (pH 7.4) supplied with 1.3 mM CaCl_2_ and 2% autologous blood plasma for 30 min at 37 °C. Each probe contained 2.3 × 10^6^ cells/mL, 1 mg/mL HSA or HSA-MG or zymosan, and 150 mM NaCl was added to control samples. * *p* < 0.05 compared to control. All measurements were performed in triplicate (*n* = 3).

Exposure of CD63 was not influenced by the incubation of neutrophils with HSA-MG compared to control (Figure 8b), which means that there was no effect of HSA or HSA-MG on the degranulation of AGs. To confirm this result, the neutrophils incubated with HSA or HSA-MG were sedimented and then lysed. Peroxidase activity in the supernatant fluid sample “1” (after cells sedimentation) and sample “2” (after cell lysis) was assessed by *o*-dianisidine oxidation, which corresponded to extracellular and intracellular activity of MPO. Then, 150 mM NaCl was added to neutrophils as a negative control and 1 mg/mL zymosan as a positive one. The results are presented in Figure 10.

HSA-MG inhibited the activity of extracellular MPO, while that of intracellular MPO remained unchanged if compared with control. This result could be interpreted as both a lack of inhibition of intracellular MPO or its release via degranulation. In preliminary experiments, it was shown that activity of extracellular MPO correlated with its concentration assessed by ELISA (using kit Cloud-Clone Corp, Houston, TX, USA: R = 0.96; *p* < 0.01; see Figure S1 in Supplementary Materials). Apparently, zymosan provoked a significant drop in intracellular MPO content, and the increase in its extracellular concentration (according to MPO activity values) resulted from the degranulation of AGs and MPO exocytosis, which is consistent with the data of other researchers [19]. The results for HSA did not differ from the control values as well as activity of intracellular MPO in the samples with HSA-MG. The activity of extracellular MPO in the latter samples was significantly reduced, in accordance with our previous results on MPO inhibition by HSA-MG (Figure 5). These data also confirm the fact that HSA-MG did not stimulate the exocytosis of MPO, and probably the degranulation of AGs.

### 3.6. Effects of HSA-MG on NETosis

**Figure 11:**
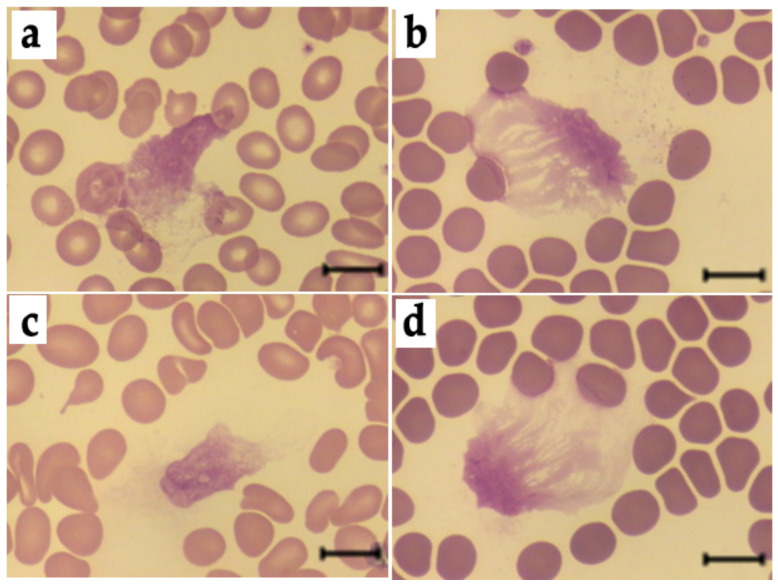
Typical examples of NET-like structures in blood smears in the presence of HSA (**a**,**b**) and HSA-MG (**c**,**d**), before (**a**,**c**) or after (**b**,**d**) the action of PMA for 2 h. Scale bar: 10 µm.

Figure 11 represents microphotographs of typical blood smears after incubation of blood with HSA or HSA-MG, where NET-like structures are clearly seen. Incubation of blood with 0.5 mg/mL or 1 mg/mL HSA-MG for 1 h or 3 h did not affect leukocyte count and NET-like structure percentage compared with HSA and control (150 mM NaCl) (Table 2). According to these data, HSA-MG did not induce NETosis under the chosen experimental conditions. Further addition of PMA to blood samples increased NET-like structure percentage and decreased leukocyte count compared to control values without PMA (Table 2), suggesting the development of NETosis. Nevertheless, preincubation neither with HSA nor with HSA-MG for 3 h affected PMA-initiated NETosis compared to control (Table 3).

## 4. Discussion

As an important component of innate immunity, neutrophils exert three major protective functions against pathogens: (i) generation of reactive bactericidal agents (ROS, RHS, reactive nitrogen species (RNS)), (ii) phagocytosis and (iii) NETosis. In the present work, we studied the effects of HSA modified under hyperglycemia-like conditions on some of these functions. First, we succeeded in demonstrating that binding of HSA-MG with MPO inhibits its peroxidase and chlorinating activity, which undoubtedly should impede the formation not only of RHS, but in some situations, that also of ROS and RNS [20,21,22]. Second, HSA-MG did not induce the degranulation of neutrophil AGs with the release of MPO—an important and necessary step for comprehensive bactericidal action. Finally, HSA-MG did not induce NETosis in whole blood and did not influence PMA-induced NETosis.

The formation of AGEs is a rather long-lasting process. In vivo, it is limited by the lifespan of a protein and can proceed for months and even for years, but in vitro, it can take some weeks to some months. Moreover, if not glucose but the natural by-product of its non-enzymatic conversion, MG, is used, the same AGEs are produced within some hours or days. Earlier, it was shown that reaction of MG with HSA is a convenient model for rather fast preparation of AGEs [9,23,24]. In our study, we used precisely this approach, incubating HSA in the presence of MG within up to 7 days. As our assays showed, all physical–chemical characteristics of the prepared HSA-MG corresponded to the parameters which were earlier reported for glycated proteins: the increase in optical absorbance at 280–320 nm and in fluorescence at 400–410 nm (excited at 325 nm) (Figure 1), aggregation and growth of negative charge (Figure 2b, lines 4 and 7). The latter can be explained, from one side, by the loss of the positive charge by amino groups, which is protonated under physiological pH values, in reaction with aldehydes; from the other side, by the formation of carbonyl compounds bearing a negative charge, for example, Nδ-carboxymethyllysine [25].

This modification of protein should increase the probability of its interaction with MPO, which is a true polycation with pI > 10 [26]. In the literature, there are numerous examples of the formation of rather stable complexes of MPO with negatively charged proteins [27,28,29], including the complex of MPO with acute-phase protein ceruloplasmin [30] which was found in the blood of patients with various inflammatory diseases [31]. It is to be noted that binding with MPO did not result in the inhibition of enzyme activity for all forms of ceruloplasmin. Breaking of one bond in the polypeptide chain could be enough for the loss of inhibitory potential of ceruloplasmin towards MPO even though the efficacy of its binding with MPO was not influenced [32]. Considering that about half of HSA-MG protein is capable of binding with MPO (Figure 3), it is not apparent that all subsets of modified albumin inhibit activity of MPO with the same efficacy.

Our results have shown that MPO forms a stable complex with negatively charged HSA-MG (K_d_ = 1.5 nM) (Figure 4). It leads to the inhibition of both peroxidase (Figure 5) and chlorinating activity (Figure 6) of MPO, but unlike ceruloplasmin, by a non-competitive mechanism. This type of inhibition is characterized by the absence of competition between HSA-MG and substrate for binding to the active site of MPO. At the same time, HSA-MG induces such changes in MPO that reduce the efficiency of conversion of the substrate to the product with no impact on the affinity of the enzyme to the substrate. Since HSA-MG competed with five various anti-MPO mAbs (Figure 4), it can be supposed that the mixture of glycation products resultant from HSA modification with MG covers a significant part of the MPO surface, thus impeding its functioning.

AGEs are capable of binding to receptors on the neutrophil surface (K_d_~3.7 ± 0.4 nM; 188 ± 5 thousand sites per cell) [6]. This binding resulted in activation of NADPH oxidase and NO-synthase and thus enhanced fMLP-stimulated production of ROS and RNS, respectively; increased the concentration of Ca^2+^ in cytoplasm driven by its efflux from intracellular stores; enhanced actin polymerization; and stimulated phagocytosis of bacteria [33,34,35]. Such neutrophil activation was accompanied by an accumulation of the products of lipid peroxidation, indicating the initiation of oxidative stress [36]. It was found that upregulation of expression and activity of phospholipase A2 is a key element in the activation of NADPH oxidase of neutrophils, the generation of superoxide anion-radical •O_2_^−^ and the induction of oxidative stress in hyperglycemia and diabetes mellitus. This enzyme hydrolyzes phospholipids at the sn-2 position to yield free fatty acids, including arachidonic acid, which stimulates NADPH oxidase assembly, thus enhancing •O_2_^−^ production [34,36].

Luc-CL is known to result mainly from the reaction of Luc with •O_2_^−^, the generation of the latter being catalyzed by NADPH oxidase [37,38]; that means that Luc-CL can be considered as a measuring instrument for NADPH oxidase activation [39]. Our results show that HSA-MG stimulates Luc-CL of neutrophils and also enhances their PMA-stimulated activation assayed by Luc-CL (Figure 7b), confirming the fact of NADPH oxidase activation by AGEs [40].

Despite the numerous data concerning the priming and activation of various signaling pathways in the cells affected by AGEs [5], there is no information on the influence of these compounds on the major neutrophil enzyme with bactericidal activity, MPO, and on the mechanisms concerning its secretion and functioning in hyperglycemia. In fact, our study is the first aimed at the elucidation of these subjects. Lum-CL of neutrophils is considered to result from oxidation of Lum by HOCl [37,41] formed in the chlorinating cycle of MPO. Hence, Lum-CL can be considered as an indicator of chlorinating activity of MPO [42]. This is confirmed by the total drop in Lum-CL of activated neutrophils caused by MPO inhibitor 4-aminobenzoic acid hydrazide, which had no effect on Luc-CL of neutrophils [43]. The addition of HSA-MG to neutrophils did not influence their Lum-CL, including their response to PMA, suggesting the inability of HSA-MG to prime and/or activate RHS generation by these cells (Figure 7a).

The analysis of HSA-MG effects on neutrophil degranulation demonstrated a dose-dependent increase in surface exposure of a marker protein of PNGs, CD11b (Figure 8). This effect of HSA-MG was prevented by ~70% by the tyrosine kinase inhibitor genistein, while the phosphoinositide-3-kinase inhibitor wortmannin inhibited CD11b exposure only by 20% (Figure 9). Thus, HSA-MG stimulates the degranulation of PNGs via a tyrosine kinase-dependent but not a phosphoinositide-3-kinase-dependent mechanism. It is worth noting that the degranulation of PNGs was equally affected by HSA modified with MG for 24 h and 7 d. Presumably, even relatively short modification resulted in the formation of compounds initiating the secretion of the content of PNGs.

At the same time, there was no significant effect of HSA-MG on the exposure of the marker protein of AGs, CD63, on the neutrophil surface (Figure 8). Apparently, HSA-MG was unable to stimulate the degranulation of AGs, including MPO secretion. This conclusion was confirmed by evaluation of the activity of intracellular MPO in neutrophils treated with HSA-MG, showing the absence of any drop in it (Figure 10).

Earlier, in equivalent experiments, we studied the effects of HSA [44,45] and low-density lipoprotein (LDL) [43,46] modified with HOCl, the major product of MPO catalysis on Luc-CL and Lum-CL, and registered the enhancement of both, accompanied by the upregulation of MPO exocytosis. We expected the same results in the present study. Nevertheless, HSA-MG neither enhanced neutrophil Lum-CL nor induced degranulation of AGs, indicating a lack of additional secretion of MPO and generation of RHS by the cells. The data obtained can be explained by considering that HOCl-modified HSA and LDL have stimulated MPO exocytosis [45,46], which apparently results in a lower probability of intracellular inactivation of MPO by ROS, whereas MPO exocytosis has not been enhanced by HSA-MG. This increases the probability of ROS-induced inactivation of MPO in the phagolysosome. Moreover, active MPO can convert ROS to HOCl which, in the phagolysosome, is rapidly scavenged by taurine, thus preventing MPO from inactivation. In the presence of HSA-MG, however, the activity of MPO is reduced and the probability of its inactivation by ROS increases, which leads to no Lum-CL increase.

One of the most important bactericidal mechanisms of neutrophil action, NETosis, consists of expelling NETs which are NET-like structures composed of decondensed chromatin with modified histones and bactericidal components of neutrophil granules, including MPO. NETosis is a way to neutralize and kill extracellular pathogens, thus minimizing risk for other cells and tissues. There are contradictory literature data on NETosis in hyperglycemia. A number of publications report activation of NETosis in hyperglycemia and diabetes mellitus [47,48]; others assert that hyperglycemia is a factor preventing NET formation in diabetes mellitus patients [49]. One way or another, our results demonstrated that HSA modified under conditions modeling hyperglycemia (treatment with MG) did not enhance NET-like structure formation in whole blood of healthy subjects and had no effect on PMA-induced NETosis (Table 2; Table 3). It was shown that MPO in NETs retains its activity, and when provided with H_2_O_2_, is able to kill *S. aureus* by a mechanism consistent with the production of HOCl [50].

It can be assumed that HSA-MG could interact with MPO associated with NETs, suppressing its bactericidal effects. The antimicrobial potential of MPO is realized also via the opsonization effects of this polycationic protein. If HSA-MG is capable of masking the sites for MPO binding with mAbs, one could expect a similar effect towards interaction of MPO with opsonins and bacterial wall components, contributing to a loss of MPO’s bactericidal activity. In diabetes mellitus type 2 patients, NETs contained less of the bactericidal protein LL-37 on their surface, which reduces their bactericidal activity [51].

In light of our current results, it becomes clear why growth in the number of bacteria phagocytized by neutrophils was accompanied by their survival in phagolysosomes, as it was shown earlier [6]. As our results have shown, HSA-MG did not promote degranulation of MPO and, probably, the release of MPO into the phagolysosome. However, even inside the phagolysosome, MPO would not effectively exert its bactericidal function since HSA-MG, binding with it, could inhibit its enzymatic activity and dampen production of bactericidal agents. Indeed, it was shown that glycated HSA also binds with lysozymes, localized in all neutrophil granules, and lactoferrin from secondary granules, inhibiting bactericidal activity of both enzymes [52]. However, for such intra-phagolysosomal effects, HSA-MG should be transported into phagolysosome, for example, in the course of phagocytoses, together with bacterial cells. Our additional experiments have shown that both non-modified HSA and HSA-MG were capable of binding to *Escherichia coli* (laboratory stain Mg1655) cells in 2.5 × 10^9^ CFU/mL suspension (up to 80 µg/mL) (data not presented). Additionally, other researchers registered binding of HSA with the cell wall of Gram-negative and Gram-positive bacteria [53]. Moreover, FITC-labeled glycated HSA was internalized in vesicles that colocalized with the lysosomal tracker (Lyso-Tracker Red DND) of neutrophils [6].

According to our data, HSA-MG, on one hand, activates NADPH-dependent production of •O_2_^−^ (Figure 7) [54], and on the other hand, inhibits chlorinating activity of MPO (Figure 6) and does not enhance its secretion by neutrophils (Figure 8; Figure 10). At the same time, hyperglycemia triggers the expression of inducible NO-synthase via activation of transcriptional nuclear factor NF-*k*B, resulting in increased generation of NO [55]. These factors enable the formation of peroxynitrite, which can affect the components of blood, provoking various effects, from stimulation of neutrophils and NETosis [56] to a decrease in platelet contractile force. Together with a drop in the clinically significant concentration of HOCl, these factors induce increased risk of thromboembolia and anormal clot retraction, impeding wound healing [57].

Coincidences of HSA-MG effects on neutrophil activity appeared in the present study, with features of neutrophils in diabetes mellitus type 2 patients assumed in Table S3 (see Supplementary Materials). In particular, NET formation, exocytosis of AGs and Lum-CL are not increased in type 2 diabetes [58,59] and are not affected by HSA-MG. On the contrary, expression of CD11b, NADPH oxidase activity and Luc-CL are increased in the case of type 2 diabetes [33,47,60,61,62,63,64] and after the addition of HSA-MG to neutrophils. Finally, the inhibition of MPO activity was described in the case of type 2 diabetes mellitus [59,65] as well as in the case of interaction HSA-MG with MPO in vitro. We can conclude that the coincidence of the effects of HSA-MG with features of neutrophil function in type 2 diabetes is hardly accidental and probably reflects the mechanisms of pathological processes leading to premature priming and activation of neutrophils and a decrease in their bactericidal activity in diabetes.

## 5. Conclusions

**Figure 12:**
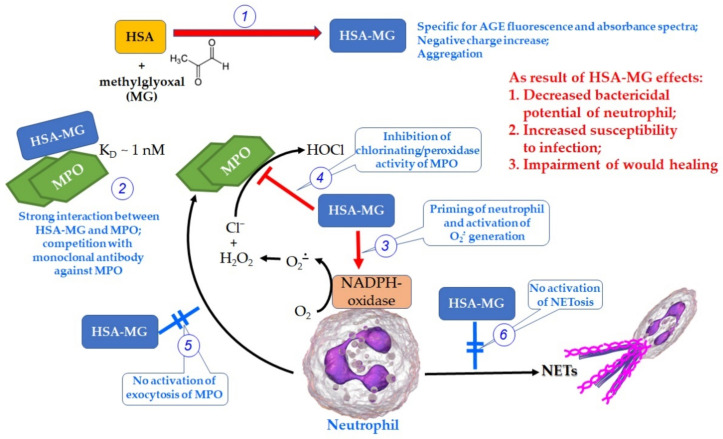
Summary scheme for the effect of HSA-MG on the function of MPO and neutrophils in the conditions of hyperglycemia modeling. Explanations are in the text.

Our main results are represented in the summarizing scheme (Figure 12).

HSA glycated by MG (reaction 1, Figure 12) forms a stable complex with MPO, inhibiting its peroxidase and its chlorinating activity (2, Figure 12). Even though HSA-MG activates neutrophils presumably via RAGE, this activation is not a full-scale one but rather one-dimensional, since mainly NADPH oxidase-dependent reactions are enhanced (3, Figure 12), consistent with numerous published data [6,33,34,35,36] and also with our analysis of Luc-CL (Figure 7b). Everything related to MPO enzymatic activity and exocytosis via degranulation or NETosis (4, 5 and 6 in Figure 12, respectively) is either not upregulated (degranulation, NETosis) or even downregulated (enzymatic activity) in the presence of HSA-MG. In perspective, the suppression of bactericidal activity of the proteins in phagolysosomes favors the expansion of infection, providing “safe refuges” for bacteria, thus protecting them from the immune system. Since high AGE concentrations were registered in most diabetes mellitus patients, reaching values up to 1.13 ± 0.14 µM [6], together with certain neutrophil dysfunction [6,66,67], a possibility exists that aberrant neutrophil reactions provoked by AGEs could underlie the neutrophil dysfunction in hyperglycemia and uncontrolled diabetes. Collectively, these factors cause the loss of immunity, delayed wound healing [68] and increased susceptibility to infection [69] in hyperglycemia and diabetes.

## Figures and Tables

**Table 1:** Kinetic parameters of peroxidase and chlorinating activity of MPO in the presence of HSA and HSA-MG ^1^.

Mixture	Parameters	Control	HSA	HSA-MG	K_i_ HSA-MG, μM
2–50 μM H_2_O_2_ (1 mM ABTS, 5 nM MPO)	K_M_, µM	21.3 ± 0.5	21.9 ± 0.6	21.5 ± 0.5	0.84
	V_max_, s^−1^	65.1 ± 1.3	64.4 ± 1.4	21.5 ± 0.5	
0.4–2 mM ABTS (50 μM H_2_O_2_, 5 nM MPO)	K_M_, mM	1.06 ± 0.08	1.05 ± 0.09	1.04 ± 0.07	1.08
	V_max_, s^−1^	87.2 ± 1.4	86.7 ± 1.5	59.7 ± 1.3	
5–80 μM H_2_O_2_ (150 mM NaCl, 10 nM MPO)	K_M_, µM	27.4 ± 1.4	26.1 ± 1.5	25.4 ± 1.3	0.80
	V_max_, s^−1^	51.2 ± 1.1	49.9 ± 1.2	31.5 ± 1.1	
25–150 mM NaCl (50 μM H_2_O_2_, 10 nM MPO)	K_M_, mM	85.7 ± 1.8	85.8 ± 1.8	85.8 ± 1.7	0.94
	V_max_, s^−1^	49.6 ± 1.0	50.2 ± 1.0	32.4 ± 0.9	

^1^ Concentration of HSA and HSA-MG was 500 nM. All measurements were performed in triplicate (*n* = 3).

**Table 2:** Effects of HSA and HSA-MG on leukocyte count and NET-like structure percentage in whole blood after 1 and 3 h incubation.

Effector	Time of Incubation, h	NET-like Structures, %	Leukocytes, Cells per µL
Control	1	7.4 ± 0.9	5300 ± 290
	3	7.1 ± 0.8	5200 ± 290
1 mg/mL HSA	1	7.4 ± 0.8	5200 ± 290
	3	7.8 ± 0.8	5000 ± 290
0.5 mg/mL HSA-MG	1	8.6 ± 0.8	5000 ± 280
	3	8.8 ± 0.9	4900 ± 260
1 mg/mL HSA-MG	1	7.2 ± 0.7	5200 ± 300
	3	7.4 ± 0.9	5200 ± 310

The data are represented as average value ± standard error of the mean (SEM) for 3–5 smears of blood from four healthy volunteers. HSA was modified with MG for 48 h.

**Table 3:** Effects of HSA and HSA-MG on leukocyte count and NET-like structure percentage in whole blood after 3 h incubation followed by addition of 100 nM PMA.

Effector	NET-like Structures, %	Leukocytes, Cells per µL
Control	12.8 ± 1.1	3900 ± 240
1 mg/mL HSA	12.1 ± 1.0	4000 ± 250
0.5 mg/mL HSA-MG	10.9 ± 1.0	4000 ± 280
1 mg/mL HSA-MG	11.8 ± 1.0	4000 ± 270

The data are represented as average value ± standard error of the mean (SEM) for 3–5 smears of blood from four healthy volunteers. HSA was modified with MG for 48 h.

## Data Availability

Data are contained within the article.

## Supplementary Materials

The following supporting information can be downloaded at: https://www.mdpi.com/article/10.3390/antiox11112263/s1, Figure S1: Correlation between peroxidase activity assessed by *o*-dianisidine test and MPO concentration assayed by ELISA in the supernatants of neutrophils incubated with various stimulators in the absence of HSA-MG. Figure S2: NET-like structures: (**a**) stained with Hoechst 33,342 dye, fluorescent microscopy; (**b**) stained with Romanowsky dye, light microscopy. Table S1: Comparison of mAbs against MPO used in study. Table S2: Characterization of ABTS and CB oxidation in the absence and presence of HSA and HSA-MG. Table S3: Comparison of neutrophil functional activity in type 2 diabetes mellitus and the effects of HSA-MG on neutrophils in vitro.

## References

[1] John W.G., Lamb E.J. (1993). The Maillard or browning reaction in diabetes. Eye.

[2] Singh K., Barden A., Mori T., Beilin L. (2001). Advanced glycation end-products: A review. Diabetologia.

[3] Angeloni C., Zambonin L., Hrelia S. (2014). Role of methylglyoxal in Alzheimer’s disease. Biomed. Res. Int..

[4] Fritz G. (2011). RAGE: A single receptor fits multiple ligands. Trends Biochem. Sci..

[5] Ott C., Jacobs K., Haucke E., Santos A.N., Grune T., Simm A. (2014). Role of advanced glycation end products in cellular signaling. Redox Biol..

[6] Collison K.S., Parhar R.S., Saleh S.S., Meyer B.F., Kwaasi A.A., Hammami M.M., Schmidt A.M., Stern D.M., Al-Mohanna F.A. (2002). RAGE-mediated neutrophil dysfunction is evoked by advanced glycation end products (AGEs). J. Leukoc. Biol..

[7] Klebanoff S.J. (2005). Myeloperoxidase: Friend and foe. J. Leukoc. Biol..

[8] Lu H., Xu S., Liang X., Dai Y., Huang Z., Ren Y., Lin J., Liu X. (2019). Advanced glycated gnd products alter neutrophil effect on regulation of CD_4_^+^ T cell differentiation through induction of myeloperoxidase and neutrophil elastase activities. Inflammation.

[9] Ahmed A., Shamsi A., Khan M.S., Husain F.M., Bilqees B. (2018). Methylglyoxal induced glycation and aggregation of human serum albumin: Biochemical and biophysical approach. Int. J. Biol. Macromol..

[10] Vakhrusheva T.V., Sokolov A.V., Kostevich V.A., Vasilyev V.B., Panasenko O.M. (2018). Enzymatic and bactericidal activity of monomeric and dimeric forms of myeloperoxidase. Biochem. Mosc. Suppl. Ser. B.

[11] Churashova I.A., Sokolov A.V., Kostevich V.A., Gorbunov N.P., Runova O.L., Firova E.M., Vasilyev V.B. (2021). Myeloperoxidase/high-density lipoprotein cholesterol ratio in patients with arterial hypertension and chronic coronary heart disease. Med. Acad. J..

[12] Sokolov A.V., Zakharova E.T., Shavlovskiĭ M.M., Vasil’ev V.B. (2005). Isolation of stable human ceruloplasmin and its interaction with salmon protamine. Bioorg. Khim..

[13] Sokolov A.V., Kostevich V.A., Kozlov S.O., Donskyi I.S., Vlasova I.I., Rudenko A.O., Zakharova E.T., Vasilyev V.B., Panasenko O.M. (2015). Kinetic method for assaying the halogenating activity of myeloperoxidase based on reaction of celestine blue B with taurine halogenamines. Free Radic. Res..

[14] Gorudko I.V., Tcherkalina O.S., Sokolov A.V., Pulina M.O., Zakharova E.T., Vasilyev V.B., Cherenkevich S.N., Panasenko O.M. (2009). New approaches to the measurement of the concentration and peroxidase activity of myeloperoxidase in human blood plasma. Russ. J. Bioorg. Chem..

[15] Basyreva L.Y., Vakhrusheva T.V., Letkeman Z.V., Maximov D.I., Fedorova E.A., Panasenko O.M., Ostrovsky E.M., Gusev S.A. (2021). Effect of vitamin D3 in combination with omega-3 polyunsaturated fatty acids on NETosis in type 2 diabetes mellitus patients. Oxid. Med. Cell. Longev..

[16] Tiruppathi C., Naqvi T., Wu Y., Vogel S.M., Minshall R.D., Malik A.B. (2004). Albumin mediates the transcytosis of myeloperoxidase by means of caveolae in endothelial cells. Proc. Natl. Acad. Sci. USA.

[17] Mócsai A., Jakus Z., Vántus T., Berton G., Lowell C.A., Ligeti E. (2000). Kinase pathways in chemoattractant-induced degranulation of neutrophils: The role of p38 mitogen-activated protein kinase activated by Src family kinases. J. Immunol..

[18] Kasper B., Brandt E., Bulfone-Paus S., Petersen F. (2004). Platelet factor 4 (PF-4)-induced neutrophil adhesion is controlled by src-kinases, whereas PF-4-mediated exocytosis requires the additional activation of p38 MAP kinase and phosphatidylinositol 3-kinase. Blood.

[19] Nøorgaard A., Andersen L.P., Nielsen H. (1995). Neutrophil degranulation by *Helicobacter pylori* proteins. Gut.

[20] Byun J., Mueller D.M., Fabjan J.S., Heinecke J.W. (1999). Nitrogen dioxide radical generated by the myeloperoxidase-hydrogen peroxide-nitrite system promotes lipid peroxidation of low density lipoprotein. FEBS Lett..

[21] Björnsdottir H., Welin A., Michaëlsson E., Osla V., Berg S., Christenson K., Sundqvist M., Dahlgren C., Karlsson A., Bylund J. (2015). Neutrophil NET formation is regulated from the inside by myeloperoxidase-processed reactive oxygen species. Free Rad. Biol. Med..

[22] Panasenko O.M., Gorudko I.V., Sokolov A.V. (2013). Hypochlorous acid as a precursor of free radicals in living systems. Biochemistry.

[23] Schmitt A., Schmitt J., Münch G., Gasic-Milencovic J. (2005). Characterization of advanced glycation end products for biochemical studies: Side chain modifications and fluorescence characteristics. Anal. Biochem..

[24] Westwood M.E., Thornalley P.J. (1995). Molecular characteristics of methylglyoxal-modified bovine and human serum albumins. Comparison with glucose-derived advanced glycation endproduct-modified serum albumins. J. Protein Chem..

[25] Silván J.M., van de Lagemaat J., Olano A., del Castillo M.D. (2006). Analysis and biological properties of amino acid derivates formed by Maillard reaction in foods. J. Pharm. Biomed. Anal..

[26] Agner K. (1941). Verdoperoxidase: A ferment isolated from neutrophils. Acta Physiol. Scand..

[27] Sokolov A.V., Pulina M.O., Ageeva K.V., Runova O.L., Zakharova E.T., Vasilyev V.B. (2007). Identification of leukocyte cationic proteins that interact with ceruloplasmin. Biochemistry.

[28] Segelmark M., Persson B., Hellmark T., Wieslander J. (1997). Binding and inhibition of myeloperoxidase (MPO): A major function of ceruloplasmin?. Clin. Exp. Immunol..

[29] Sokolov A.V., Ageeva K.V., Pulina M.O., Cherkalina O.S., Samygina V.R., Vlasova I.I., Panasenko O.M., Zakharova E.T., Vasilyev V.B. (2008). Ceruloplasmin and myeloperoxidase in complex affect the enzymatic properties of each other. Free Radic. Res..

[30] Samygina V.R., Sokolov A.V., Bourenkov G., Petoukhov M.V., Pulina M.O., Zakharova E.T., Vasilyev V.B., Bartunik H., Svergun D.I. (2013). Ceruloplasmin: Macromolecular assemblies with iron-containing acute phase proteins. PLoS ONE.

[31] Sokolov A.V., Pulina M.O., Ageeva K.V., Ayrapetov M.I., Berlov M.N., Volgin G.N., Markov A.G., Yablonsky P.K., Kolodkin N.I., Zakharova E.T. (2007). Interaction of ceruloplasmin, lactoferrin, and myeloperoxidase. Biochemistry.

[32] Zabrodskaya Y.A., Egorov V.V., Sokolov A.V., Shvetsov A.V., Gorshkova Y.E., Ivankov O.I., Kostevich V.A., Gorbunov N.P., Ramsay E.S., Fedorova N.D. (2022). Caught red handed: Modeling and confirmation of the myeloperoxidase ceruloplasmin alpha-thrombin complex. Biometals.

[33] Wong R.K.M., Pettit A.I., Davies J.E., Ng L.L. (2002). Augmentation of the neutrophil respiratory burst through the action of advanced glycation end products. Diabetes.

[34] Wong R.K.M., Pettit A.I., Quinn P.A., Jennings S.C., Davies J.E., Ng L.L. (2003). Advanced glycation end products stimulate an enhanced neutrophil respiratory burst mediated through the activation of cytosolic phospholipase A2 and generation of arachidonic acid. Circulation.

[35] Bansal S., Siddarth M., Chawla D., Banerjee B.D., Madhu S.V., Tripathi A.K. (2012). Advanced glycation end products enhance reactive oxygen and nitrogen species generation in neutrophils in vitro. Mol. Cell. Biochem..

[36] Ayilavarapu S., Kantarci A., Fredman G., Turkoglu O., Omori K., Liu H., Iwata T., Yagi M., Hasturk H., van Dyke T.E. (2010). Diabetes-induced oxidative stress is mediated by Ca^2+^-independent phospholipase A2 in neutrophils. J. Immunol..

[37] Vladimirov Y.A., Proskurnina E.V. (2009). Free radicals and cell chemiluminescence. Biochemistry.

[38] Li Y., Zhu H., Kuppusamy P., Roubaud V., Zweier J.L., Trush M.A. (1998). Validation of lucigenin (bis-N-methylacridinium) as a chemilumigenic probe for detecting superoxide anion radical production by enzymatic and cellular systems. J. Biol. Chem..

[39] Afanas’ev I.B., Ostrakhovitch E.A., Mikhal’chik E.V., Korkina L.G. (2001). Direct enzymatic reduction of lucigenin decreases lucigenin-amplified chemiluminescence produced by superoxide ion. Luminescence.

[40] Mikhalchik E.V., Ivanov V.A., Borodina I.V., Pobeguts O.V., Smirnov I.P., Gorudko I.V., Grigorieva D.V., Boychenko O.P., Moskalets A.P., Klinov D.V. (2022). Neutrophil activation by mineral microparticles coated with methylglyoxal-glycated albumin. Int. J. Mol. Sci..

[41] Brestel E.P. (1985). Co-oxidation of luminol by hypochlorite and hydrogen peroxide implications for neutrophil chemiluminescence. Biochem. Biophys. Res. Commun..

[42] Panasenko O.M., Chekanov A.V., Vlasova I.I., Sokolov A.V., Ageeva K.V., Pulina M.O., Cherkalina O.S., Vasil’ev V.B. (2008). Influence of ceruloplasmin and lactoferrin on the chlorination activity of leukocyte myeloperoxidase assayed by chemiluminescence. Biophysics.

[43] Panasenko O.M., Mikhalchik E.V., Gorudko I.V., Grigorieva D.V., Sokolov A.V., Kostevich V.A., Vasilyev V.B., Cherenkevich S.N. (2016). The effects of antioxidants and hypohalous acid scavengers on neutrophil activation by hypochlorous acid-modified low-density lipoproteins. Biophysics.

[44] Mikhalchik E.V., Smolina N.V., Astamirova T.S., Gorudko I.V., Grigorieva D.V., Ivanov V.A., Sokolov A.V., Kostevich V.A., Cherenkevich S.N., Panasenko O.M. (2013). Human serum albumin modified under oxidative/halogenative stress enhances luminal-dependent chemiluminescence of human neutrophils. Biophysics.

[45] Gorudko I.V., Grigorieva D.V., Shamova E.V., Kostevich V.A., Sokolov A.V., Mikhalchik E.V., Cherenkevich S.N., Arnhold J., Panasenko O.M. (2014). Hypohalous acid-modified human serum albumin induces neutrophil NADPH oxidase activation, degranulation, and shape change. Free Radic. Biol. Med..

[46] Sokolov A.V., Kostevich V.A., Runova O.L., Gorudko I.V., Vasilyev V.B., Cherenkevich S.N., Panasenko O.M. (2014). Proatherogenic modification of LDL by surface-bound myeloperoxidase. Chem. Phys. Lipids.

[47] Mikhalchik E.V., Lipatova V.A., Basyreva L.Y., Panasenko O.M., Gusev S.A., Sergienko V.I. (2021). Hyperglycemia and some aspecrs of leukocyte activation in vitro. Bull. Exp. Biol. Med..

[48] Berezin A. (2019). Neutrophil extracellular traps: The core player in vascular complications of diabetes mellitus. Diabetes Metab. Syndr..

[49] Joshi M.B., Lad A., Prasad A.S.B., Balakrishnan A., Ramachandra L., Satyamoorthy K. (2013). High glucose modulates IL-6 mediated immune homeostasis through impeding neutrophil extracellular trap formation. FEBS Lett..

[50] Parker H., Albrett A.M., Kettle A.J., Winterbourn C.C. (2012). Myeloperoxidase associated with neutrophil extracellular traps is active and mediates bacterial killing in the presence of hydrogen peroxide. J. Leukoc. Biol..

[51] Arampatzioglou A., Papazoglou D., Konstantinidis T., Chrysanthopoulou A., Mitsios A., Angelidou I., Maroulakou I., Ritis K., Skendros P. (2018). Clarithromycin enhances the antibacterial activity and wound healing capacity in type 2 diabetes mellitus by increasing LL-37 load on neutrophil extracellular traps. Front. Immunol..

[52] Li Y.M., Tan A.X., Vlassara H. (1995). Antibacterial activity of lysozyme and lactoferrin is inhibited by binding of advanced glycation-modified proteins to a conserved motif. Nat. Med..

[53] Dalhoff A. (2018). Seventy-five years of research on protein binding. Antimicrob. Agents Chemother..

[54] Pacher P., Beckman J.S., Liaudet L. (2007). Nitric oxide and peroxynitrite in health and disease. Physiol. Rev..

[55] Cosentino F., Hishikawa K., Katusic Z.S., Luscher T.F. (1997). High glucose increases nitric oxide synthase expression and superoxide anion generation in human aortic endothelial cells. Circulation.

[56] Manda-Handzlik A., Bystrzycka W., Cieloch A., Glodkowska-Mrowka E., Jankowska-Steifer E., Heropolitanska-Pliszka E., Skrobot A., Muchowicz A., Ciepiela O., Wachowska M. (2020). Nitric oxide and peroxynitrite trigger and enhance release of neutrophil extracellular traps. Cell. Mol. Life Sci..

[57] Misztal T., Rusak T., Tomasiak M. (2014). Clinically relevant HOCl concentrations reduce clot retraction rate via the inhibition of energy production in platelet mitochondria. Free Radic. Res..

[58] You Q., He D.M., Shu G.F., Cao B., Xia Y.Q., Xing Y., Ni M., Chen J.F., Shi S.L., Gu H.F. (2019). Increased formation of neutrophil extracellular traps is associated with gut leakage in patients with type 1 but not type 2 diabetes. J. Diabetes.

[59] De Souza Ferreira C., Araújo T.H., Ângelo M.L., Pennacchi P.C., Okada S.S., de Araújo Paula F.B., Migliorini S., Rodrigues M.R. (2012). Neutrophil dysfunction induced by hyperglycemia: Modulation of myeloperoxidase activity. Cell Biochem. Funct..

[60] Senior P.A., Marshall S.M., Thomas T.H. (1999). Dysregulation of PMN antigen expression in Type 2 diabetes may reflect a generalized defect of exocytosis: Influence of hypertension and microalbuminuria. J. Leukoc. Biol..

[61] Advani A., Marshall S.M., Thomas T.H. (2002). Impaired neutrophil actin assembly causes persistent CD11b expression and reduced primary granule exocytosis in Type II diabetes. Diabetologia.

[62] Mastej K., Adamiec R. (2008). Neutrophil surface expression of CD11b and CD62L in diabetic microangiopathy. Acta Diabetol..

[63] Hand W.L., Hand D.L., Vasquez Y. (2007). Increased polymorphonuclear leukocyte respiratory burst function in type 2 diabetes. Diabetes Res. Clin. Pract..

[64] Omori K., Ohira T., Uchida Y., Ayilavarapu S.D., Batista E.L., Yagi M., Iwata T., Liu H., Hasturk H., Kantarci A. (2008). Priming of neutrophil oxidative burst in diabetes requires preassembly of the NADPH oxidase. J. Leukoc. Biol..

[65] Unubol M., Yavasoglu I., Kacar F., Guney E., Omurlu I.K., Ture M., Kadikoylu G., Bolaman Z. (2015). Relationship between glycemic control and histochemical myeloperoxidase activity in neutrophils in patients with type 2 diabetes. Diabetol. Metab. Syndr..

[66] Shah S.V., Wallin J.D., Eilen S.D. (1983). Chemiluminescence and superoxide anion production by leukocytes from diabetic patients. J. Clin. Endrocrinol. Metab..

[67] Sawant J.K. (1993). Biochemical changes in polymorphonuclear leucocytes in diabetic patients. J. Postgrad. Med..

[68] Rosenberg C.S. (1990). Wound healing in the patient with diabetes mellitus. Nurs. Clin. N. Am..

[69] Mancini L., Ruotolo V. (1997). Infection of the diabetic foot. Rays.

