# Supplementary material for: Methylglyoxal-Modified Human Serum Albumin Binds to Leukocyte Myeloperoxidase and Inhibits its Enzymatic Activity
Source: Antioxidants (Basel). 2022 Nov 16;11(11):2263. doi: 10.3390/antiox11112263 (PMC9686918; doi:10.3390/antiox11112263)
Supplement: Supplementary file 1 [file antioxidants-11-02263-s001.zip › antioxidants-1947233-supplementary.pdf]

Supplementary data

Methylglyoxal-Modified Human Serum Albumin Binds to Leukocyte Myeloperoxidase and Inhibits its Enzymatic Activity

Oleg M. Panasenko [1,2,*], Viktor A. Ivanov [1], Elena V. Mikhalchik [1], Irina V. Gorudko [3], Daria V. Grigorieva [3], Liliya Yu. Basyreva [1], Ekaterina V. Shmeleva [1], Sergey A. Gusev [1], Valeria A. Kostevich [1,4], Nikolay P. Gorbunov [1,4] and Alexey V. Sokolov [1,4]

1 Department of Biophysics, Federal Research and Clinical Center of Physical-Chemical Medicine of Federal Medical Biological Agency, Moscow 119435, Russia
2 Department of Medical Biophysics of the Institute for Translative Medicine, Pirogov Russian National Research Medical University, Moscow 117997, Russia
3 Department of Biophysics, Belarusian State University, 220030 Minsk, Belarus
4 Department of Molecular Genetics, Institute of Experimental Medicine, St. Petersburg 197376, Russia
* Correspondence: o-panas@mail.ru; Tel.: +7-499-246-44-90

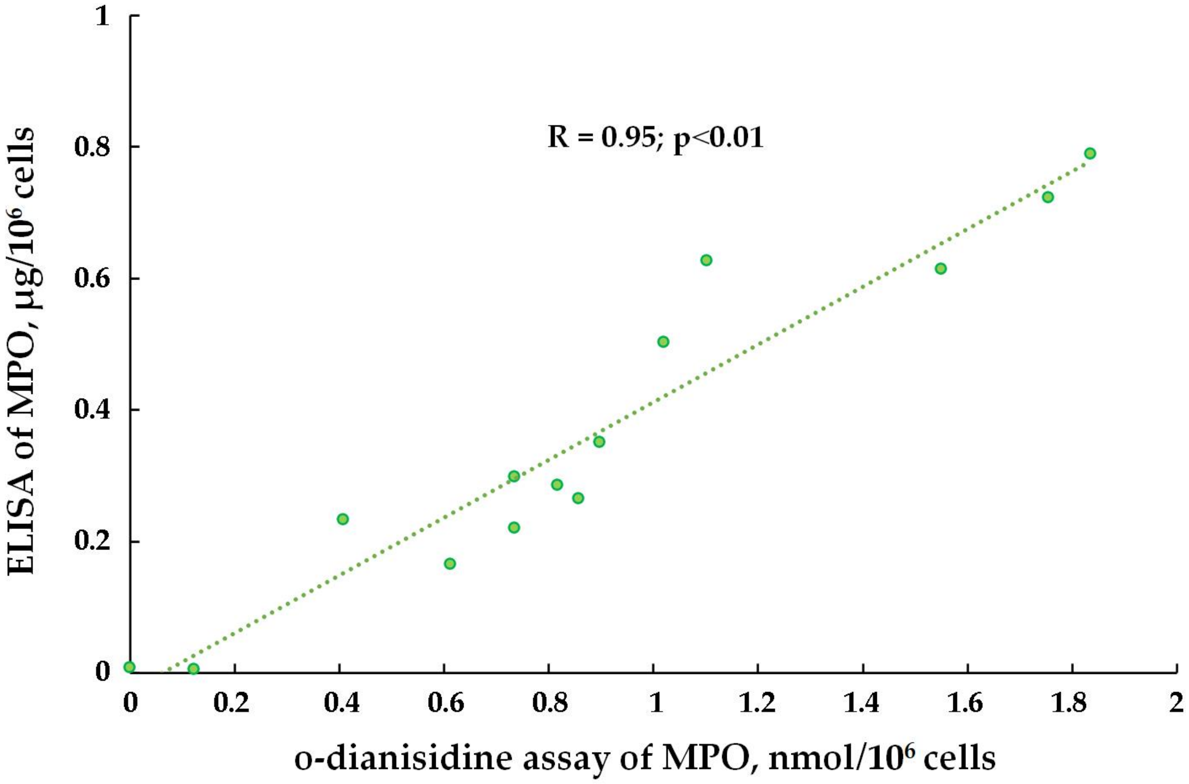

Figure S1. Correlation between MPO peroxidase activity assessed by o-dianisidine test and MPO concentration assayed by ELISA in the supernatants of neutrophils incubated with stimulators in the absence of HSA-MG.

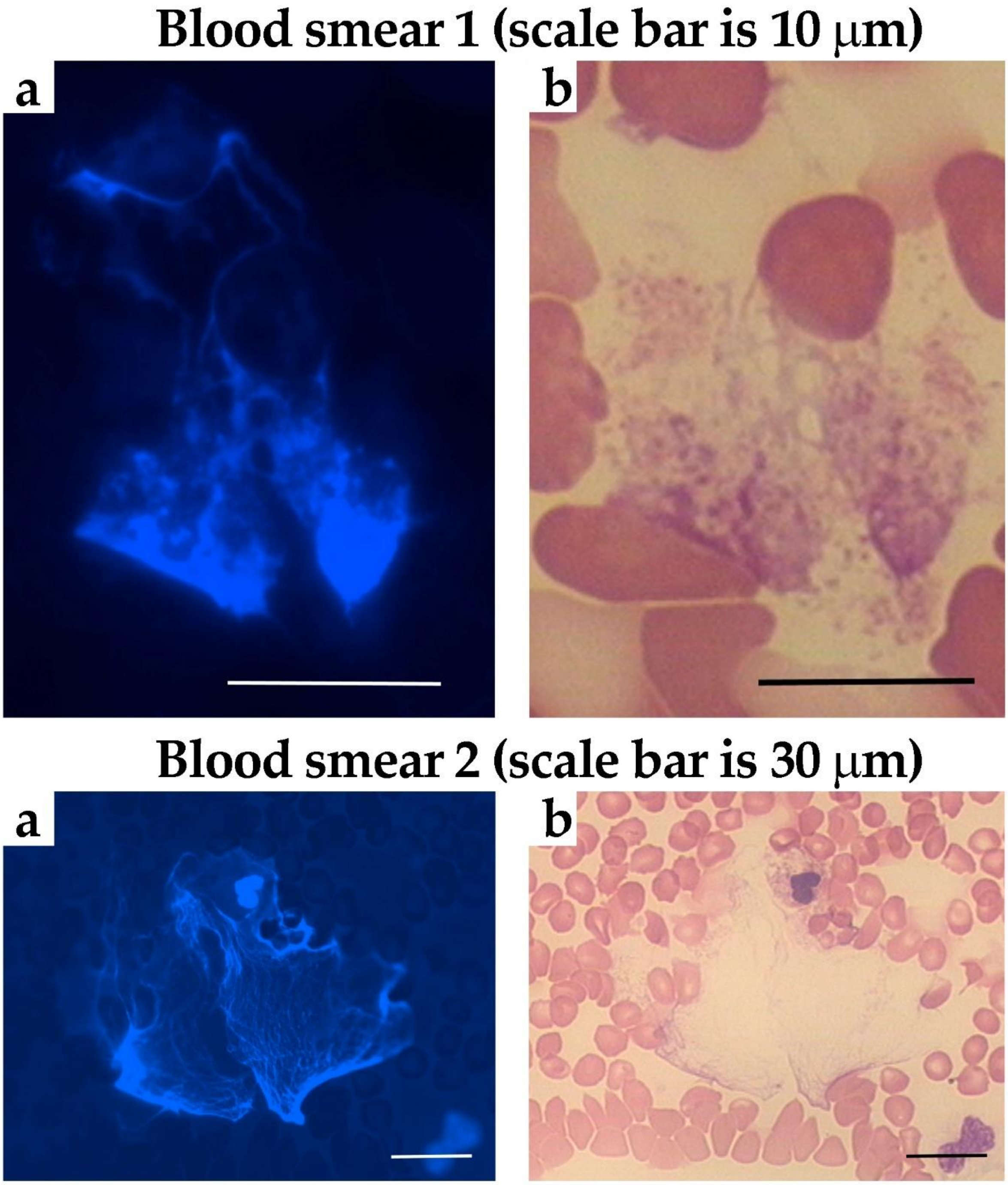

Figure S2. NET-like structures: (a) stained with Hoechst 33342 dye, fluorescent microscopy; (b) stained with Romanowsky dye, light microscopy.

Table S1. Comparison of mAbs against MPO used in study

mAb	Working combination for hemi-MPO detection with HRP-labeled mAb (tested mAb on solid phase)	Detection of dimeric MPO with same HRP-labeled mAb (tested mAb on solid phase)	Resistance of interaction with MPO to 2 M NaCl
1#8	2#7-HRP<br>4#2-HRP<br>4#4-HRP<br>4#8-HRP<br>4#10-HRP	+	+
2#7	1#8-HRP<br>4#2-HRP<br>4#4-HRP<br>4#8-HRP<br>4#10-HRP	+	-
4#2	1#8-HRP<br>2#7-HRP<br>4#4-HRP<br>4#8-HRP<br>4#10-HRP	+	-
4#4	1#8-HRP<br>2#7-HRP<br>4#2-HRP<br>4#4-HRP<br>4#10-HRP	No detection	-
4#8	1#8-HRP<br>2#7-HRP<br>4#2-HRP<br>4#4-HRP<br>4#10-HRP	+	-
4#10	No working combinations	No detection	+

Note: For unambiguous characterisation of epitope specificity of solid-phase immobilized and labeled mAbs we used hemi-MPO and not MPO dimer, due to the fact that results obtained with MPO dimer in sandwich ELISA cannot be interpreted in a unique manner (in terms of epitope specificity of mAbs).

Table S2. Comparison of ABTS and CB oxidation in absence and presence of HSA and HSA-MG (n = 3)

Reaction mixture	**ABTS oxidation – A_{414}/min** (1 mM ABTS, 5 nM HRP, 2 mM H_2O_2, 50 mM Na-acetate buffer, pH 5.5)	**CB oxidation – μM HOCl** (200 μM CB, 25 μM HOCl, 150 mM NaCl, 2 mM taurine, 10 μM KI, 20 mM Na-phosphate buffer, pH 5.8)
**Control**	0.996 ± 0.031	24.8 ± 0.4
**500 nM HSA**	1.019 ± 0.042	25.1 ± 0.3
**500 nM HSA-MG**	1.004 ± 0.029	24.9 ± 0.4

Table S3. Comparison of neutrophil functional activity in type 2 diabetes mellitus and the effects of HSA-MG on neutrophils in vitro (see articles [1–9])

Functional activity of neutrophil	Type 2 diabetes mellitus	HSA-MG (this study)
Expression of CD11b	Increased [1–3]	Increased
Expression of CD63, exocytosis of AGs	No effect [4]	No effect
MPO chlorinating activity	Decreased [5,6]	Inhibition
Lum-CL	No effect [5]	No effect
Luc-CL, NADPH-oxidase activity	Increased [6–9]	Increased
NETosis	No effect [4]	No effect

References

1. Senior, P.A.; Marshall, S.M.; Thomas, T.H. Dysregulation of PMN antigen expression in Type 2 diabetes may reflect a generalized defect of exocytosis: Influence of hypertension and microalbuminuria. *J. Leukoc. Biol*. **1999**, *65*, 800–807. https://doi.org/10.1002/jlb.65.6.800.
2. Advani, A.; Marshall, S.M.; Thomas, T.H. Impaired neutrophil actin assembly causes persistent CD11b expression and reduced primary granule exocytosis in Type II diabetes. *Diabetologia* **2002**, *45*, 719–727. https://doi.org/10.1007/s00125-002-0802-0.
3. Mastej, K.; Adamiec, R. Neutrophil surface expression of CD11b and CD62L in diabetic microangiopathy. *Acta Diabetol.* **2008**, *45*, 183. https://doi.org/10.1007/s00592-008-0040-0.
4. You, Q.; He, D.M.; Shu, G.F.; Cao, B.; Xia, Y.Q.; Xing, Y.; Ni, M.; Chen, J.F.; Shi, S.L.; Gu, H.F.; et al. Increased formation of neutrophil extracellular traps is associated with gut leakage in patients with type 1 but not type 2 diabetes. *J. Diabetes* **2019**, *11*, 665–673. https://doi.org/10.1111/1753-0407.12892.
5. De Souza Ferreira, C.; Araújo, T.H.; Ângelo, M.L.; Pennacchi, P.C.; Okada, S.S.; de Araújo Paula, F.B.; Migliorini, S.; Rodrigues, M.R. Neutrophil dysfunction induced by hyperglycemia: Modulation of myeloperoxidase activity. *Cell Biochem. Funct*. **2012**, *30*, 604–610. https://doi.org/10.1002/cbf.2840.
6. Unubol, M.; Yavasoglu, I.; Kacar, F.; Guney, E.; Omurlu, I.K.; Ture, M.; Kadikoylu, G.; Bolaman, Z. Relationship between glycemic control and histochemical myeloperoxidase activity in neutrophils in patients with type 2 diabetes. *Diabetol. Metab. Syndr.* **2015**, *7*, 119. https://doi.org/10.1186/s13098-015-0115-3.
7. Wong, R.K.M.; Pettit, A.I.; Davies, J.E.; Ng, L.L. Augmentation of the neutrophil respiratory burst through the action of advanced glycation end products. *Diabetes* **2002**, *51*, 2846–2853. https://doi.org/10.2337/diabetes.51.9.2846.
8. Mikhalchik, E.V.; Lipatova, V.A.; Basyreva, L.Y.; Panasenko, O.M.; Gusev, S.A.; Sergienko, V.I. Hyperglycemia and some aspecrs of leukocyte activation in vitro. *Bull. Experim. Biol. Med*. **2021**, *170*, 748–751. https://doi.org/10.1007/s10517-021-05147-x.
9. Omori, K.; Ohira, T.; Uchida, Y.; Ayilavarapu, S.D.; Batista, E.L., Jr.; Yagi, M.; Iwata, T.; Liu, H.; Hasturk, H.; Kantarci, A.; et al. Priming of neutrophil oxidative burst in diabetes requires preassembly of the NADPH oxidase. *J. Leukoc. Biol.* **2008**, *84*, 292–301. https://doi.org/10.1189/jlb.1207832.
